# A near complete, chromosome-scale assembly of the black raspberry (*Rubus occidentalis*) genome

**DOI:** 10.1093/gigascience/giy094

**Published:** 2018-08-09

**Authors:** Robert VanBuren, Ching Man Wai, Marivi Colle, Jie Wang, Shawn Sullivan, Jill M Bushakra, Ivan Liachko, Kelly J Vining, Michael Dossett, Chad E Finn, Rubina Jibran, David Chagné, Kevin Childs, Patrick P Edger, Todd C Mockler, Nahla V Bassil

**Affiliations:** 1 Department of Horticulture, Michigan State University, East Lansing, MI, 48824, USA; 2 Plant Resilience Institute, Michigan State University, East Lansing, MI, 48824, USA; 3 Department of Plant Biology, Michigan State University, East Lansing, MI, 48824, USA; 4 Phase Genomics, Seattle, WA, 98195, USA; 5 USDA-ARS National Clonal Germplasm Repository, 33447 Peoria Rd., Corvallis, OR, 97333, USA; 6 Blueberry Council (in Partnership with Agriculture and Agri-Food Canada) Agassiz Food Research Centre, BC V0M 1A0, Canada; 7 USDA-ARS Horticultural Crops Research Unit, Corvallis, OR 97330, USA; 8 The New Zealand Institute for Plant & Food Research Limited, Private Bag 11600, Palmerston North 4474, New Zealand; 9 The Donald Danforth Plant Science Center, St. Louis, MO 63132, USA

**Keywords:** Plant genetics, Genome assembly, Comparative genomics

## Abstract

**Background:**

The fragmented nature of most draft plant genomes has hindered downstream gene discovery, trait mapping for breeding, and other functional genomics applications. There is a pressing need to improve or finish draft plant genome assemblies.

**Findings:**

Here, we present a chromosome-scale assembly of the black raspberry genome using single-molecule real-time Pacific Biosciences sequencing and high-throughput chromatin conformation capture (Hi-C) genome scaffolding. The updated V3 assembly has a contig N50 of 5.1 Mb, representing an ∼200-fold improvement over the previous Illumina-based version. Each of the 235 contigs was anchored and oriented into seven chromosomes, correcting several major misassemblies. Black raspberry V3 contains 47 Mb of new sequences including large pericentromeric regions and thousands of previously unannotated protein-coding genes. Among the new genes are hundreds of expanded tandem gene arrays that were collapsed in the Illumina-based assembly. Detailed comparative genomics with the high-quality V4 woodland strawberry genome (*Fragaria vesca*) revealed near-perfect 1:1 synteny with dramatic divergence in tandem gene array composition. Lineage-specific tandem gene arrays in black raspberry are related to agronomic traits such as disease resistance and secondary metabolite biosynthesis.

**Conclusions:**

The improved resolution of tandem gene arrays highlights the need to reassemble these highly complex and biologically important regions in draft plant genomes. The updated, high-quality black raspberry reference genome will be useful for comparative genomics across the horticulturally important Rosaceae family and enable the development of marker assisted breeding in *Rubus*.

## Introduction

To date, more than 200 plant genomes have been sequenced, including most plants with agronomic value. Notable exceptions include large, polyploid, or otherwise complex genomes and many horticultural, medicinal, or orphan crop species [1]. Most plant genomes were assembled using short-read (50–500 bp), next-generation sequencing (NGS)-based approaches such as Illumina and 454 pyrosequencing technologies. The low cost and high throughput of NGS technologies facilitated rapid genomic resource development, but the short read lengths produced low-quality assemblies compared to the early Sanger-based plant genomes [1]. NGS-based assemblies contain gaps in repetitive regions that exceed the maximum read lengths, and most genomes have thousands to millions of imbedded sequence gaps. These gaps can span biologically important sequences including tandem gene arrays, repeat dense, and haplotype- or homeologous-specific regions. Recent advances in single-molecule real-time (SMRT) sequencing have overcome the previous limitations of NGS-based approaches and ushered in a new era of “platinum-quality” reference genomes [2]. The long read lengths of Pacific Biosciences (PacBio)- and Nanopore-based SMRT sequencing allow accurate assembly and phasing of complex genomic regions. SMRT sequencing has been used to drastically improve the contiguity of the maize [3], apple [4], woodland strawberry [5], and rice genomes [6], among others.

Black raspberry (*Rubus occidentalis* L.) is an important specialty fruit crop in the US Pacific Northwest that is closely related to the globally commercialized red raspberry (*Rubus idaeus* L.). Black raspberry has undergone little improvement since its domestication in the late 1800s [7], and elite cultivars suffer from limited genetic diversity [8, 9]. Genomic resources for *Rubus* are needed to accelerate marker-assisted selection and improvement. The black raspberry genome was sequenced using an NGS-based approach, yielding a fragmented but much needed draft assembly [10]. This draft was anchored into a chromosome-scale assembly using a high-throughput chromatin conformation capture (Hi-C)-based scaffolding approach [11]. However, the reference used for scaffolding is ∼50 Mb smaller than the estimated genome size and is likely missing important genomic features. Here, we utilized long-read PacBio sequencing and Hi-C to finish and re-annotate the black raspberry genome. The updated V3 reference is nearly complete and includes thousands of new genes, making it useful for the plant comparative genomics and *Rubus* breeding communities.

## Results

**Figure 1:**
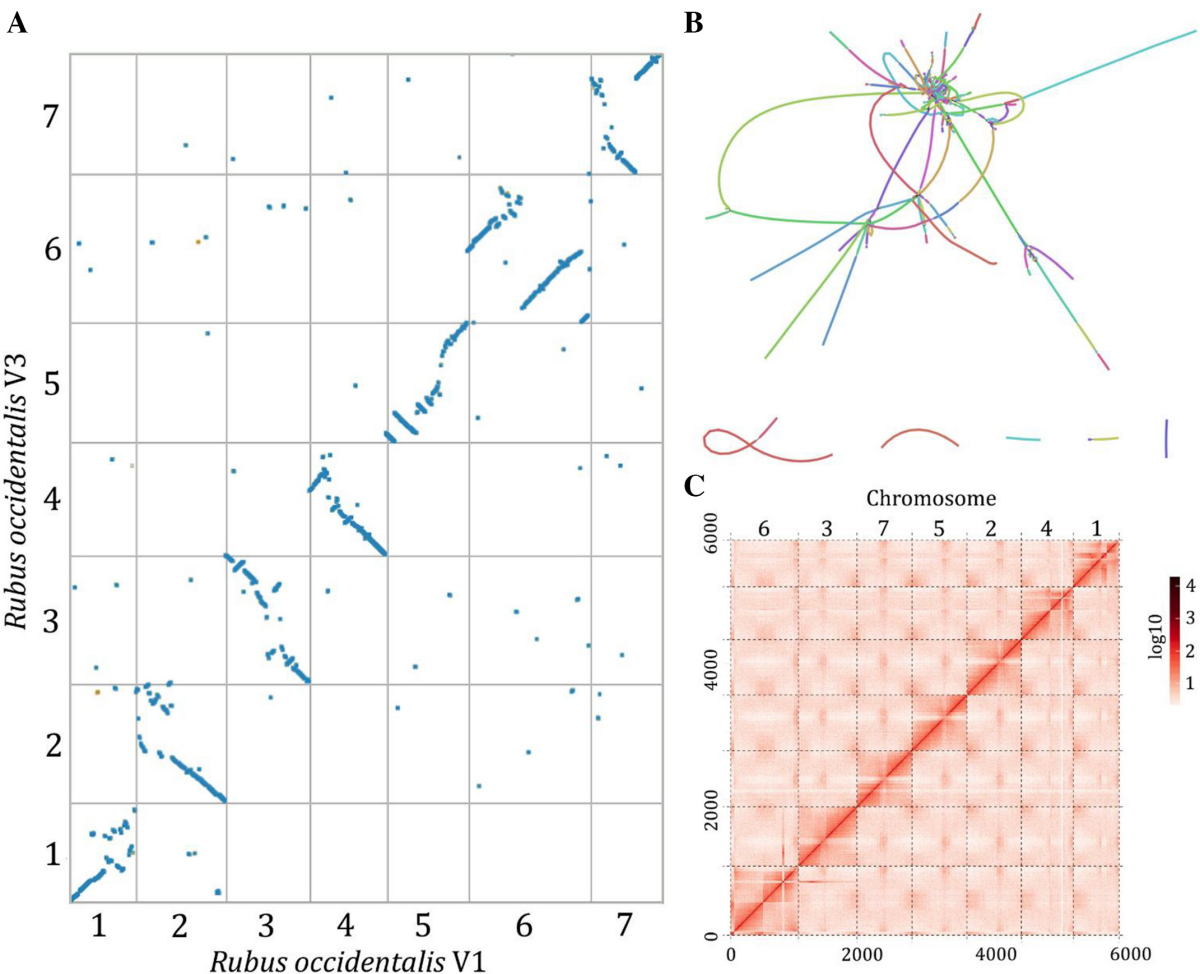
Updated chromosome scale assembly of black raspberry. **(A)** Syntenic dot plot of the black raspberry V1 and V3 assemblies. Each blue point denotes a collinear genomic region. **(B)** Assembly graph of the V3 reference. Each line (node) represents a contig in the Canu assembly, and connections (edges) between contigs represent ambiguities in the graph structure. The color of contigs is randomly assigned. **(C)** Post-clustering heat map showing density of Hi-C interactions between contigs from the Proximity-Guided Assembly.

To improve the black raspberry reference genome, we generated 2.1 million PacBio reads collectively spanning 21.8 Gb or 76x genome coverage. The PacBio data have a subread N50 length of 11.5 kb, average length of 9.8 kb, and maximum length of 72 kb (Supplementary Fig. S1). PacBio reads shorter than 1 kb were discarded, and reads longer than 10 kb were used as seeds for error correction and assembly using the Canu assembler [12]. The Canu-based assembly was improved by two rounds of polishing with Pilon [13] using high-coverage (∼80x) paired-end Illumina data to correct residual insertion/deletion errors. The final assembly has a contig N50 of 5.1 Mb across 235 contigs and total size of 290 Mb (Table1). This represents a ∼200x improvement in contiguity compared to the Illumina-only assembly and includes more than 47 Mb of additional sequences. Newly assembled sequences consist of mostly repetitive elements but also include regions containing protein coding genes (described below). The Canu assembly graph is free of bubbles associated with heterozygous regions, but there is some graph complexity resulting from high copy number repetitive elements (Fig.1B).

**Table 1:** Comparison of the black raspberry V1 and V3 assemblies

	V1	V3
Total assembly size, Mb	243	290
Number of contigs	11,936	235
Number of scaffolds	2,226	7
Contig N50, kb	33.1	5100
Scaffold N50, Mb	0.35	41.1
LTR composition, %	16.20	32.60
Number of genes	28,005	34,545

The PacBio-based contigs were assembled into scaffolds and then into pseudomolecules using Hi-C and Proximity-Guided Assembly. This approach was previously used by Jibran et al. [11] to cluster and order 9,650 of the 11,936 V1 black raspberry contigs into seven pseudomolecules spanning 223.8 Mb (97.3% of the assembly). The Illumina-based Hi-C data from Jibran et al. [11] was remapped to the PacBio assembly and clustered into seven pseudomolecules using the Proximo Hi-C scaffolding pipeline (Fig. 1C). The Hi-C scaffolding was able to anchor and order with high confidence all 235 contigs into seven pseudomolecules with sizes ranging from 34 Mb to 51 Mb with an N50 of 41.1 Mb (Table2). The pseudomolecules were assigned to the seven haploid black raspberry chromosomes (Ro01–Ro07) using markers from sequence-based genetic maps as anchors [14]. We used PBJelly [15] to fill gaps in the pseudomolecules with error-corrected PacBio reads exceeding 10 kb in length. This approach successfully filled 16 of the 228 gaps; the remaining gaps are likely either complex or highly repetitive with nonunique junctions exceeding read lengths.

**Table 2:** Summary of chromosome anchoring using the HiC genome map

Chromosome	Anchored contigs	Total size (bp)
Ro01	19	34,302,027
Ro02	19	40,757,823
Ro03	30	43,767,452
Ro04	30	38,746,748
Ro05	25	41,095,993
Ro06	37	50,854,034
Ro07	75	41,277,220
Total	235	290,801,297

**Figure 2:**
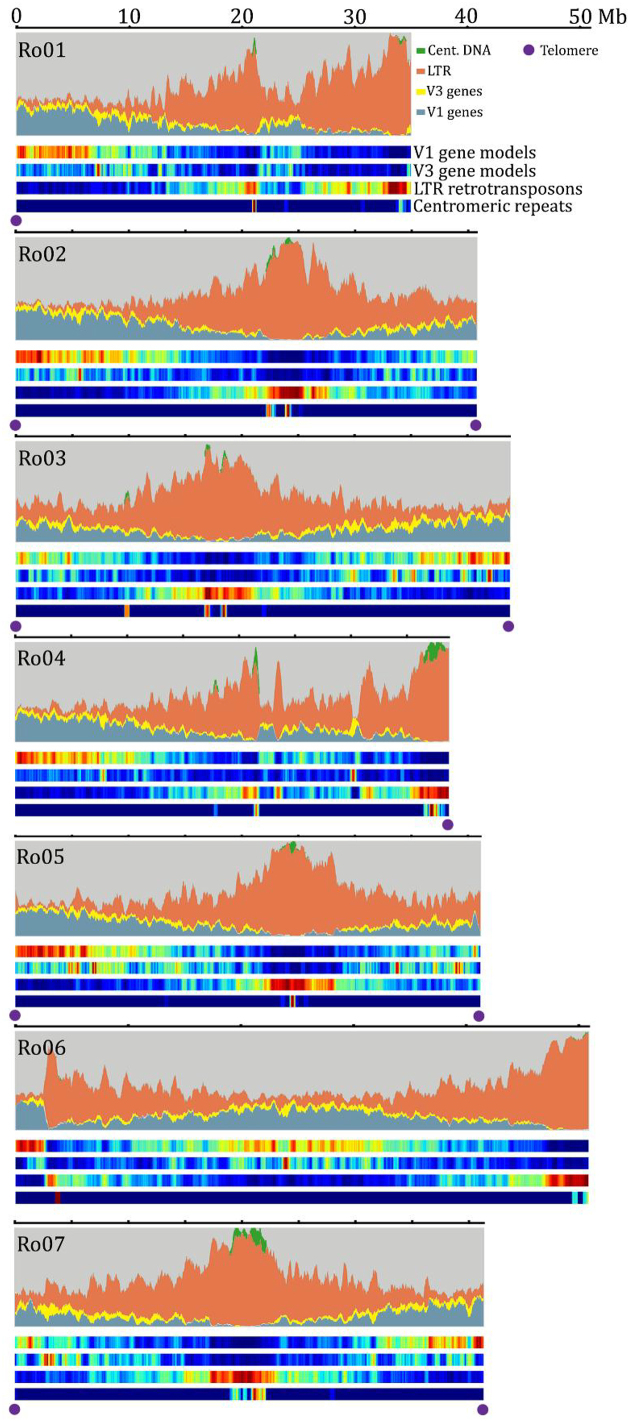
Genome landscape of the black raspberry V3 genome. The composition of long terminal repeat (LTR) retrotransposons, centromeric repeat arrays (Cent. DNA), gene models carried over from the V1 assembly, and new gene models in V3 are plotted in 50-kb bins with a 25-kb sliding window. Terminal telomeric repeats are denoted by purple dots.

The combined PacBio and Hi-C assembly (hereafter referred to as V3) contains 10 terminal telomeric tracks at both ends of chromosomes Ro02, Ro03, Ro05, and Ro07 and at one end of Ro01 and Ro04, validating the accuracy and quality of our assembly (Fig.2). We identified a novel 317-bp centromeric repeat with high abundance in six of the seven chromosomes. Centromeric repeat array sizes range from 110 elements in Ro01 to 1,204 in Ro04, with element homologies averaging 89% (Supplementary Table S1). The presence of centromeric arrays, repetitive element density, and Hi-C-based intrachromosomal interactions allowed us to estimate the centromere size in each chromosome. Black raspberry chromosomes have an average centromere size of 2.8 Mb, with individual sizes ranging from 173 kb in Ro01 to 5.2 Mb in Ro03. Ro06 contained only four centromeric repeats with no obvious enrichment of repetitive elements or reduction in intrachromosomal interactions based on the Hi-C data, suggesting the centromeric region of this chromosome is still largely unassembled. The proportion of long terminal repeat (LTR) retrotransposons in the black raspberry genome nearly doubled, with an increase from 16.2% in V1 to 32.6% in V3. Intact LTR retrotransposons are a metric for assembly quality, and the number of intact elements increased from 258 in V1 to 2,342 in V3. LTR and gene density are inversely correlated, with pericentromeric and subtelomeric regions having the highest LTR density (Fig.2). Together, the accurate assembly of highly repetitive regions and relatively low number of remaining sequence gaps suggest the V3 black raspberry assembly is nearly complete.

We aligned the V3 black raspberry assembly to the V1 pseudomolecules to assess genome collinearity. We identified numerous misassembles in V1 spanning most of the genome (Fig. 1A). Misassembled regions range from small-scale inversions reflecting incorrect scaffold orientation to major chromosome arm-sized inversions in Ro06 and Ro07. The pericentromeric regions are largely unassembled in V1, resulting in large gaps in the syntenic dot plots. Major gaps are also found throughout genic regions in the genome. The errors in V1 likely stem from read length limitations of NGS data and errors in marker order from the genetic maps that were used to build the pseudomolecules. A similar level of scaffold misassembly was observed in the comparison of PacBio-based V4 woodland strawberry genome to the previous Illumina-based genome [5]. Such errors are probably common in most NGS-based plant genomes and are hindering marker-assisted breeding efforts.

**Figure 3:**
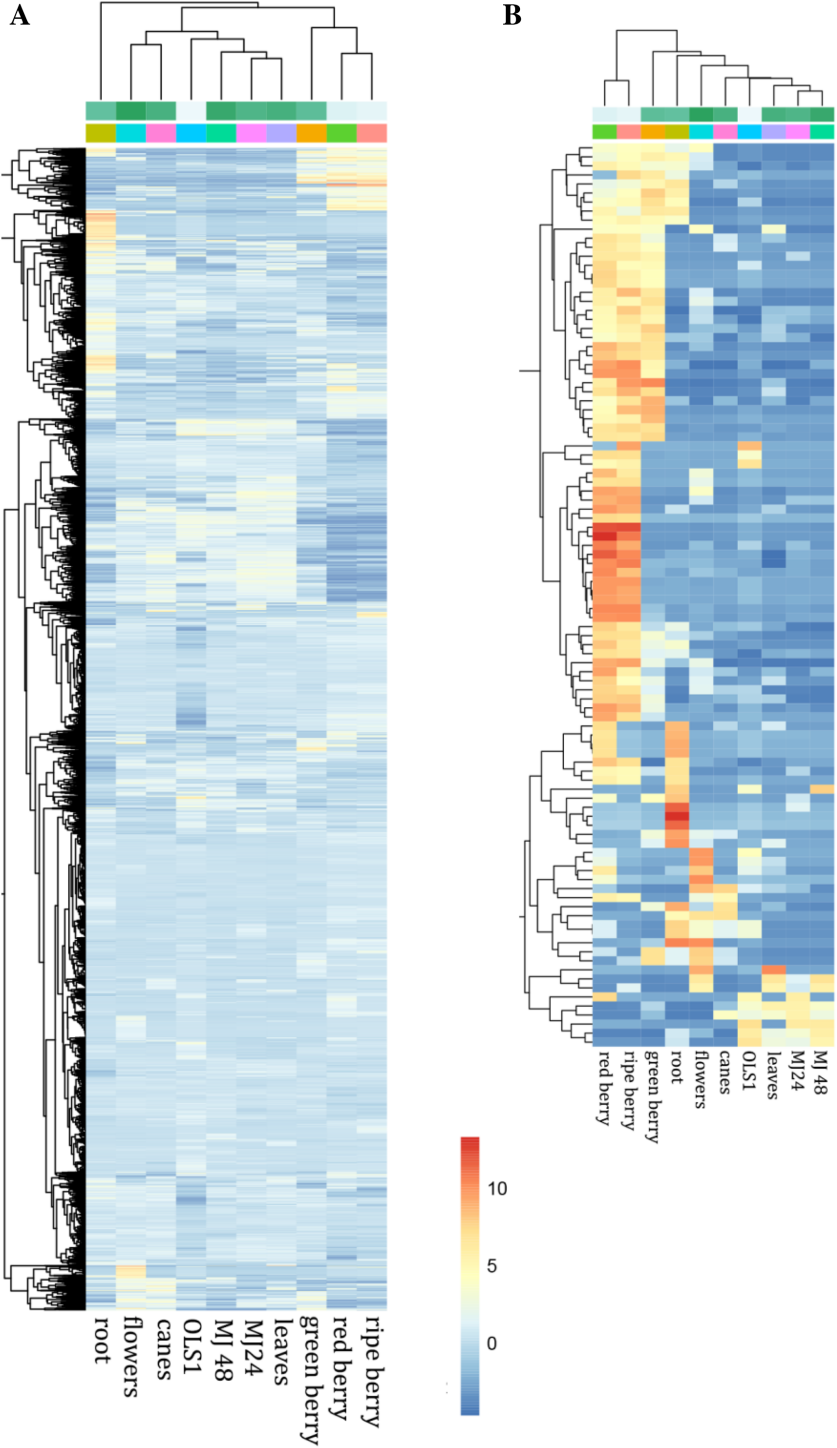
Expression patterns of new genes in the V3 black raspberry assembly. **(A)** Heat map of expression patterns of all 6,070 new genes with detectable expression. **(B)** Expression patterns of the top 100 genes with highest expression. Blue indicates low expression and red indicates high expression. Expression values are plotted as log2 transformed FPKM.

The V3 black raspberry assembly includes 43 Mb of new sequences that were unassembled in the V1 reference. We re-annotated the V3 assembly *ab initio* using the MAKER-P pipeline [16]. Ten RNA sequencing (RNA-seq) datasets from a diverse tissue atlas were assembled with StringTie [17] and used as transcript evidence. Gene models from the diploid strawberry (*Fragaria vesca*) [5] and Arabidopsis (TAIR10) [18] genomes were used as protein evidence. The new annotation has 34,545 high-confidence gene models, substantially more than the 28,005 models in the V1 assembly. We assessed annotation quality using the Benchmarking Universal Single-Copy Orthologs [19] pipeline and found 94% (1,352 of 1,440) of the genes in the embryophyta dataset present in the V3 assembly, compared to 87% in the V1 black raspberry reference. This proportion is similar to other recent PacBio-based genomes [2, 5, 20] and suggests the annotation is of high quality. The V3 annotation includes 9,301 new gene models that were improved or absent from the V1 assembly; 4,020 low-quality gene models from V1 were removed in V3. The discarded gene models had insufficient transcript or protein support or transposable element–related annotations. The average gene length is 3,165 bp and 3,220 bp for V1 and V3, respectively, suggesting residual indels in V3 are not resulting in fragmented gene models. Most of the newly annotated genes (6,070 out of 9,301) have detectable expression (fragments per kilobase of transcript per million mapped reads [FPKM] >1) in the gene expression atlas figures (Fig. 3). Many new genes have tissue-specific expression patterns (Supplementary Fig. S2), which may explain why they were missed in the V1 annotation.

**Figure 4:**
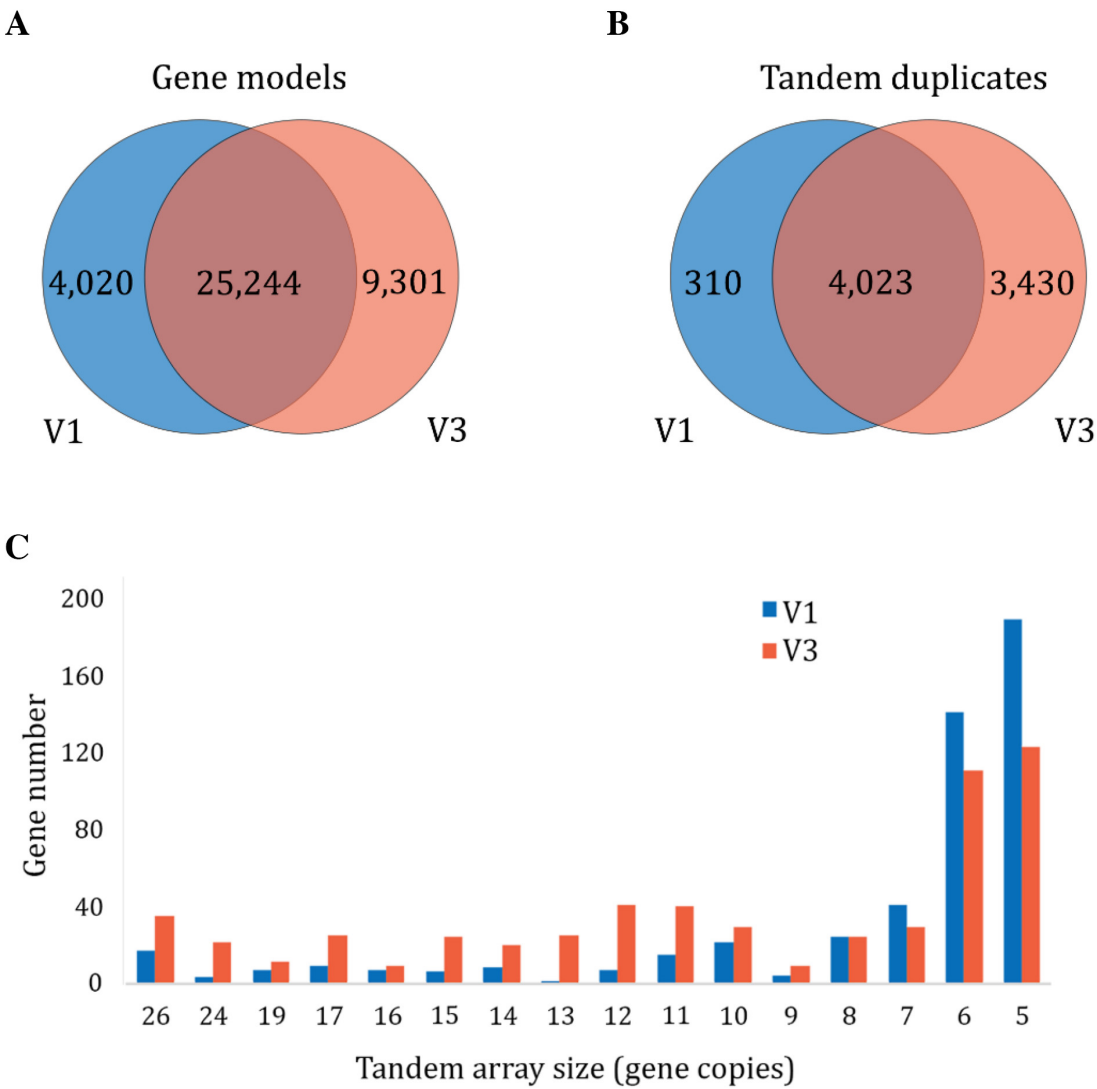
Comparison of tandem gene array sizes in the V1 and V3 black raspberry assemblies. **(A)** Venn diagram of gene models specific to V1 (blue), specific to V3 (orange), and shared. **(B)** Comparison of total tandem gene duplicates in V1 and V3. **(C)** The number of genes found in both the V1 and V3 assemblies (blue) or only V3 (orange) is plotted for tandem arrays ranging in size from 5 to 26 copies. Array size is based on the V3 annotation.

The V3 black raspberry annotation has a striking increase in the size and number of tandem gene arrays. Tandem gene duplicates (TDs) with high sequence homology often collapse into single gene copies during the assembly of NGS data and are likely underrepresented in most genomes. We identified 7,453 TDs in the V3 assembly compared to 4,333 in V1. Tandem arrays range in size from 2 to 26 copies, with an average size of 4. Large tandem arrays show the greatest improvement in assembly accuracy, with the most dramatic increase from four copies in V1 to 26 in V3 (Fig.4). Tandem arrays with more than 10 genes have, on average, 52% more annotated copies in V3, while tandem arrays with 5–9 genes have, on average, 31% more annotated copies in V3. Most arrays with two or three TDs are unchanged in the V3 assembly; 16% of arrays were completely novel, with no homology to gene models in V1. Some differences in tandem array length are likely due to improvements in the annotation.

**Figure 5:**
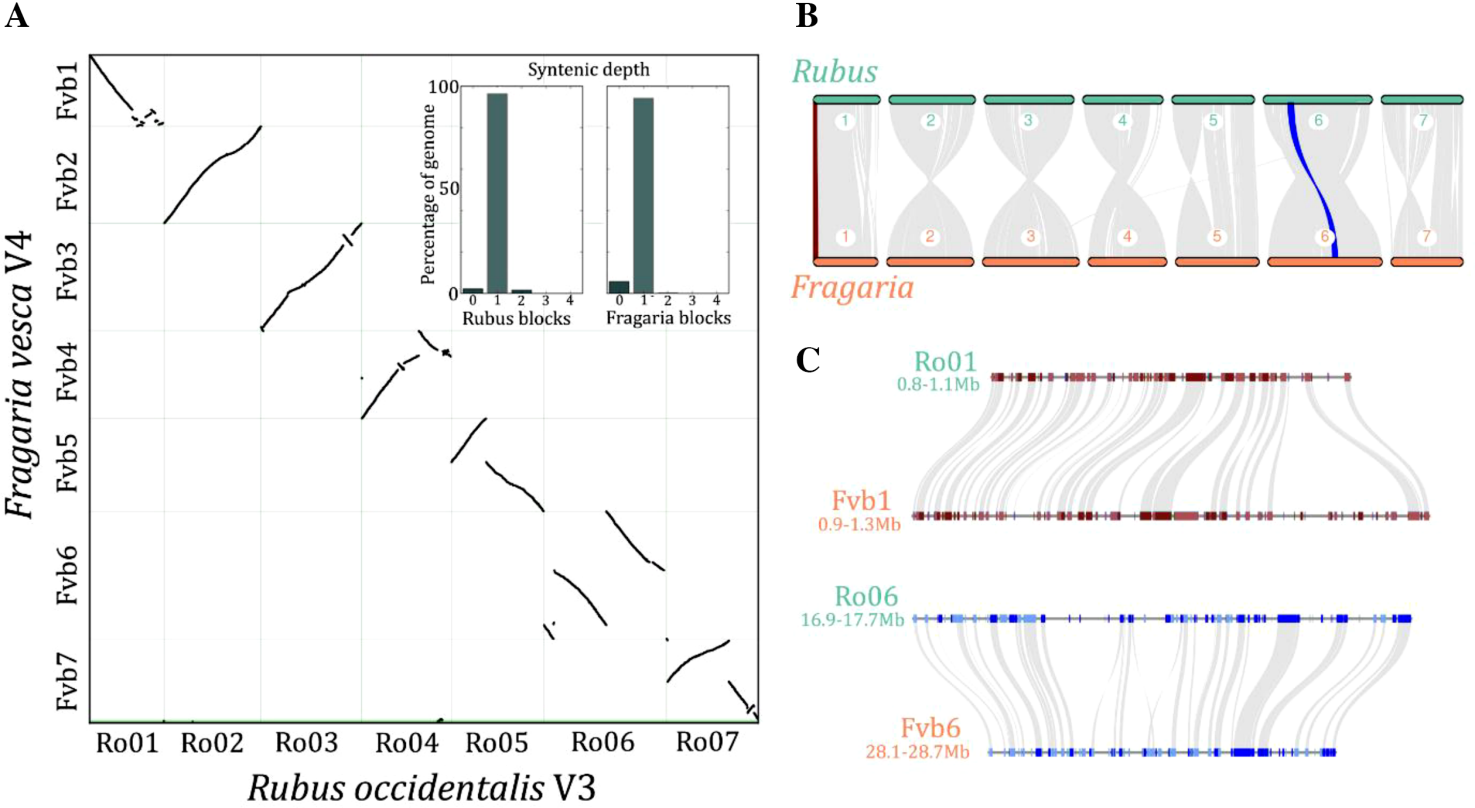
Comparative genomics of the black raspberry V3 and woodland strawberry (*Fragaria vesca*) V4 genomes. **(A)** Macrosyntenic dot plot between the black raspberry and *F. vesca* genomes. Each black dot represents a syntenic region between the two genomes. The inlaid bar graph shows syntenic depth of each red raspberry and *F. vesca* syntenic block. **(B)** Chromosome-scale collinearity between black raspberry and *F. vesca*. Collinear regions between chromosomes Ro01 and Fvb1 and between chromosomes Ro06 and Fvb6 are highlighted in red and blue respectively and shown in more detail in **(C)**. **(C)** Microsynteny of two regions showing lineage-specific expansion in Fvb1 (top comparison) and Ro06 (bottom). Genes are shown in red or blue (top and bottom, respectively), with colors indicating gene orientation (light are forward, dark are reverse). Syntenic gene pairs are connected by gray lines.

Black raspberry is in the Rosaceae, a large and diverse family that includes peach, pear, apple, strawberry, cherry, plum, rose, and almonds among other important horticultural crops. Genomes are available for many of these crop species, providing an excellent framework for comparative functional genomic analyses. The closest crop relatives of black raspberry are the cultivated strawberries (*Fragaria* sp.), with the most common recent ancestor of these two species having diverged ∼75 million years ago [21]. Woodland strawberry (*F. vesca*) and black raspberry have the same karyotype (2*n* = 14), and previous genetic map and genomic analyses suggest a high degree of collinearity [10]. We utilized the PacBio-based V4 *F. vesca* assembly [5] to make detailed comparisons between these two species. Despite the 75 million year divergence, the black raspberry and *F. vesca* genomes are largely collinear (Fig. 5). Ro01/Fvb1, Ro02/Fvb2, and Ro03/Fvb3 have no major structural rearrangements, and the other four chromosome pairs have one or two major inversions (Fig.5A). Surprisingly, there are no translocations between chromosomes in either species. More than 96% of collinear blocks have 1:1 syntenic depth with no large-scale segmental duplications. The black raspberry and *F. vesca* genomes have 15,727 syntenic gene pairs, which is consistent with other similarly diverged lineages such as species within Poaceae [2, 22]. The black raspberry and *F. vesca* genomes are similar in size (290 vs 240 Mb, respectively), and each genome has unique patterns of expansion/deletion based on gene-level microsynteny (Fig.5C). Differences in gene composition between species are likely due to a combination of tandem gene duplications, retrotransposon-mediated duplication/movement, fractionation/deletion, and misannotation. Expansion/deletion outside of genic regions is likely related to differences in repetitive element composition. We identified 615 syntenic tandem gene arrays that are conserved between *F. vesca* and black raspberry and 1,231 that are unique in either species. Syntenic TDs range in copy number, and no TDs with more than three copies have the same array size in both species. Most of the lineage-specific syntenic TDs have two or three copies, but we identified 16 arrays with more than 10 copies in black raspberry and only 1 copy in *F. vesca* (Supplementary Table S2).

The drastic improvements in the V3 black raspberry genome will help accelerate marker-assisted selection and functional genomics studies in *Rubus*. Most of the newly assembled black raspberry sequences are repetitive, but other collapsed regions such as tandem gene arrays were also drastically improved. Gene duplications drive evolutionary innovation [23], and these regions likely underlie important domestication- and improvement-related traits. The black raspberry gene expression atlas also helped improve the structure of several thousand existing and new gene models that were incorrectly annotated or missing due to a lack of transcript support. Numerous scaffolding errors were fixed in V3 and the corrected pseudomolecules will facilitate more accurate marker ordering and fine mapping for quantitative trait loci (QTL) and genome wide association studies (GWAS). Black raspberry joins a growing list of near-complete, reference-grade genome assemblies for the plant comparative genomics community.

## Methods

### DNA extraction and genome assembly

High-molecular-weight (HMW) genomic DNA (gDNA) was isolated from young leaf tissue of black raspberry selection ORUS 4115-3 using a modified nuclei preparation method [24]. A 20-kb insert library was constructed from the HMW gDNA followed by size selection on the BluePippin (Safe Science) and sequencing on a PacBio RSII platform using P6-C4 chemistry. Raw PacBio reads were corrected and assembled using the Canu assembler V1.4 (Canu, RRID:SCR_015880) [12]. The following parameters were modified: minReadLength = 2,000, GenomeSize = 290 Mb, minOverlapLength = 1,000. Other parameters were left as defaults. The PacBio-based contigs were polished with Pilon V1.22 (Pilon, RRID:SCR_014731) [13] using ∼80x Illumina paired-end data from the V1 black raspberry draft genome assembly [10]. Quality-trimmed Illumina reads were aligned to the draft PacBio-based contigs using bowtie2 (V2.3.0) [25] with default parameters. The alignment rate of Illumina data was ∼98%, supporting the completeness of our assembly. Illumina reads were realigned around insertions/deletions using the IndelRealigner function from the genome analysis tool kit V3.7 (GATK, RRID:SCR_001876) [26]. The parameters for Pilon were as follows: –flank 7, –K 49, and –mindepth 20. Pilon was run a second time using the polished contigs as a reference to correct any residual errors. After two rounds of polishing, 106,929 indels and 2,900 single nucleotide polymorphisms were corrected in the assembly. Since the assembly was not polished using the PacBio reads, it is possible long-range misassemblies were missed by Pilon and may still be present in our assembly.

### Pseudomolecule construction and validation

Hi-C library construction and sequencing was previously reported [11]. In total, 54.4 million Hi-C read pairs from Jibran et al. [11] were generated and used as input to the Proximo Hi-C scaffolding pipeline. Reads were aligned to the polished PacBio contigs using BWA V0.7.16 (BWA, RRID:SCR_010910) [27] with strict parameters (-n 0) to prevent mismatches and nonspecific alignments. Only read pairs that aligned to different contigs were used for scaffolding. The Proximo Hi-C pipeline performed chromosome clustering and contig orientation as described previously [28]. Briefly, Proximo utilizes an enhanced version of the LACHESIS algorithm as well as scaffold optimization and extra quality-control steps to group and orient contigs based on interaction probabilities. Hi-C interactions binned the contigs into seven groups (corresponding to the haploid chromosomes) and successfully oriented all 235 contigs. The gap length between ordered contigs was set at 100 bp. Pseudomolecules were assigned to chromosomes using simple sequence repeat (SSR)- and genotyping by sequencing (GBS)- based markers from high-density genetic maps [14]. Gaps in the pseudomolecules were filled using error-corrected PacBio reads with PBJelly V 15.8.24 (PBJelly, RRID:SCR_012091) [15] using default parameters. This near-complete version has been designated as V3.

### Identification of centromeric regions

Centromeres were identified using three lines of evidence: reduced intrachromosomal interactions in the Hi-C heat map, increased density of LTR retrotransposons, and presence of centromere-specific tandem repeat arrays (317 bp). First, the intrachromosomal interactions were used to locate the putative centromere locations. Centromere locations were validated by overlap with centromere-specific tandem repeat arrays. The estimated borders of centromeres were identified by the presence of centromere-specific tandem repeats and LTR retrotransposon density >85%. Putative centromeres were identified for six of the seven black raspberry chromosomes, and no enrichment of centromere tandem repeats was found in Ro06. Centromere-specific repeat arrays were found in only nine reads in the unassembled read file from Canu, suggesting the centromeres are well assembled in black raspberry.

### Genome annotation

The MAKER-P pipeline (MAKER, RRID:SCR_005309) [16] was used to annotate the V3 assembly. Ten RNA-seq datasets (described below) used as transcript evidence and gene models from the diploid strawberry (*F. vesca*) [5] and Arabidopsis (TAIR10) [18] genomes were used as protein evidence. The RNA-seq samples were assembled into transcripts using a reference-guided approach with StringTie (V1.3.3) [17]. A custom LTR retrotransposon library was created using the LTR_retriever pipeline [29]. This custom library was used in conjunction with the MAKER repeat library for masking prior to annotation. *Ab initio* gene prediction was performed using SNAP and Augustus (Augustus: Gene Prediction, RRID:SCR_008417), with three and two rounds of reiterative training, respectively. The resulting gene set was filtered to remove gene models containing Pfam domains related to transposable elements, resulting in an annotation of 33-286 gene models. Annotation quality was assessed using the Benchmarking Universal Single-Copy Orthologs V3 (BUSCO, RRID:SCR_015008) [19] pipeline with the embryophyta dataset of 1,440 single-copy conserved genes.

### Expression analysis

To build a gene expression atlas, RNA was collected from 10 diverse black raspberry tissues. This includes green berries, red berries, ripe berries, flowers, canes, roots, leaves, and methyl jasmonate-treated leaf tissue. Fresh tissue was flash-frozen in liquid nitrogen, and total RNA was extracted using KingFisher Pure RNA Plant kit (Thermo Fisher Scientific, MA), according to the manufacturer's instructions. Two micrograms of total RNA was used to construct stranded mRNA libraries (KAPA mRNA HyperPrep kit, KAPA Biosystems, Roche, USA). Multiplexed, pooled libraries were sequenced on the Illumina HiSeq4000 under paired-end  150-nt mode in the genomics core at Michigan State University. Raw reads were trimmed using Trimmomatic V 0.33 (Trimmomatic, RRID:SCR_011848) [30] and aligned to the black raspberry V3 genome using the STAR aligner [31]. Reads were then assembled using a reference-guided approach with StringTie (V1.3.3) [17] and output as read count tables. Expression analyses were performed using the DESeq2 pipeline [32] and visualized using the pheatmap R package [33]. Tissue-specific expression was defined as having >1 FPKM in one tissue and FPKM <1 in all other tissues.

### Comparative genomics

The black raspberry V3 genome was compared to the black raspberry V1 [10] and *F. vesca* V4 [5] genomes using the MCScan toolkit (V1.1) [34]. Syntenic gene pairs were identified using all vs all Basic Local Alignment Search Tool followed by filtering for 1:1 collinear pairs with MCScan. Tandem gene duplicates were identified using a minimum e-value of 1^e-5^ and maximum gene distance of 10 genes. Pair-wise, macrosynteny, and microsynteny plots were constructed using the python version of MCScan [35].

## Supplementary Material

GIGA-D-18-00032_Original_Submission.pdf

GIGA-D-18-00032_Revision_1.pdf

Response_to_Reviewer_Comments_Original_Submission.pdf

Reviewer_1_Report_(Original_Submission) -- Felix Bemm 2/26/2018 Reviewed

Reviewer_1_Report_(Revision_1) -- Felix Bemm 5/15/2018 Reviewed

Reviewer_2_Original_Submission_(attachment).pdf

Reviewer_2_Report_(Original_Submission) -- Susan Strickler 3/14/2018 Reviewed

Supplemental Tables and Figures

## Availability of supporting data

The updated black raspberry V3 assembly and annotation can be downloaded from CoGe (https://genomeevolution.org/coge) under Genome ID 37 280 and the genome database for Rosaceae (https://www.rosaceae.org/). The raw sequence data have been deposited in the Short Read Archive (SRA) under NCBI BioProject ID PRJNA430858. Supporting data are also available via the GigaDB dataset for this project [36].

## Abbreviations

Hi-C: high-throughput chromatin confirmation capture; NGS: next generation sequencing; SMRT: single molecule real-time; LTR: long terminal repeat; FPKM fragments per kilobase of transcripts per million mapped reads; TD: tandem gene duplicate; QTL: quantitative trait loci; GWAS: genome wide association study; SSR: simple sequence repeat; GBS: genotyping by sequencing

## References

[1] Michael TP, VanBuren R Progress, challenges and the future of crop genomes. Curr Opin Plant Biol. 2015;24:71–81. 25703261 10.1016/j.pbi.2015.02.002

[2] VanBuren R, Bryant D, Edger PP Single-molecule sequencing of the desiccation-tolerant grass *Oropetium thomaeum*. Nature. 2015;527(7579):508–11. 26560029 10.1038/nature15714

[3] Jiao Y, Peluso P, Shi J, Improved maize reference genome with single-molecule technologies. Nature. 2017;546:524–7. 28605751 10.1038/nature22971 PMC7052699

[4] Daccord N, Celton J-M, Linsmith G, High-quality de novo assembly of the apple genome and methylome dynamics of early fruit development. Nat Genet. 2017;49:1099–106. 28581499 10.1038/ng.3886

[5] Edger PP, VanBuren R, Colle M Single-molecule sequencing and optical mapping yields an improved genome of woodland strawberry (*Fragaria vesca*) with chromosome-scale contiguity. GigaScience. 2017;7:1–7. 10.1093/gigascience/gix124 PMC5801600 29253147

[6] Du H, Yu Y, Ma Y Sequencing and de novo assembly of a near complete indica rice genome. Nature Communications. 2017;8:15324. 10.1038/ncomms15324 PMC5418594 28469237

[7] Jennings DL Raspberries and blackberries: their breeding, diseases and growth. Academic Press;.

[8] Dossett M, Bassil NV, Lewers KS, Genetic diversity in wild and cultivated black raspberry (*Rubus occidentalis*L.) evaluated by simple sequence repeat markers. Genetic Resources and Crop Evolution. 2012;59(8):1849–65.

[9] Dossett M, Lee J, Finn CE Inheritance of phenological, vegetative, and fruit chemistry traits in black raspberry. J Am Soc Hortic Sci. 2008;133(3):408–17.

[10] VanBuren R, Bryant D, Bushakra JM The genome of black raspberry (*Rubus occidentalis*). Plant J. 2016;87(6):535–47. 27228578 10.1111/tpj.13215

[11] Jibran R, Dzierzon H, Bassil N Chromosome-scale scaffolding of the black raspberry (*Rubus occidentalis* L.) genome based on chromatin interaction data. Horticulture Research. 2018;5(1):8. 29423238 10.1038/s41438-017-0013-y PMC5802725

[12] Koren S, Walenz BP, Berlin K Genome Research. 2017;27(5): 722–736. 10.1101/gr.215087.116 PMC5411767 28298431

[13] Walker BJ, Abeel T, Shea T Pilon: an integrated tool for comprehensive microbial variant detection and genome assembly improvement. PLoS One. 2014;9(11):e112963. 25409509 10.1371/journal.pone.0112963 PMC4237348

[14] Finn CE, Lee J, VanBuren R A genetic linkage map of black raspberry (*Rubus occidentalis*) and the mapping of Ag (4) conferring resistance to the aphid *Amphorophora agathonica*. Theor Appl Genet. 2015;128:1631–46. 26037086 10.1007/s00122-015-2541-x PMC4477079

[15] English AC, Richards S, Han Y Mind the gap: upgrading genomes with Pacific Biosciences RS long-read sequencing technology. PLoS One. 2012;7(11):e47768. 23185243 10.1371/journal.pone.0047768 PMC3504050

[16] Campbell MS, Law M, Holt C MAKER-P: a tool kit for the rapid creation, management, and quality control of plant genome annotations. Plant Physiol. 2014;164(2):513–24. 24306534 10.1104/pp.113.230144 PMC3912085

[17] Pertea M, Pertea GM, Antonescu CM StringTie enables improved reconstruction of a transcriptome from RNA-seq reads. Nat Biotechnol. 2015;33(3):290–5. 25690850 10.1038/nbt.3122 PMC4643835

[18] Lamesch P, Berardini TZ, Li D, The Arabidopsis Information Resource (TAIR): improved gene annotation and new tools. Nucleic Acids Res. 2011;40(D1):D1202–D10. 22140109 10.1093/nar/gkr1090 PMC3245047

[19] Simão FA, Waterhouse RM, Ioannidis P BUSCO: assessing genome assembly and annotation completeness with single-copy orthologs. Bioinformatics. 2015;31(19):3210–2. 26059717 10.1093/bioinformatics/btv351

[20] Jarvis DE, Ho YS, Lightfoot DJ The genome of *Chenopodium quinoa*. Nature. 2017;542(7641):307–12. 28178233 10.1038/nature21370

[21] Xiang Y, Huang C-H, Hu Y, Evolution of Rosaceae fruit types based on nuclear phylogeny in the context of geological times and genome duplication. Mol Biol Evol. 2016;34(2):262–81. 10.1093/molbev/msw242 PMC5400374 27856652

[22] Paterson AH, Bowers JE, Bruggmann R, The sorghum bicolor genome and the diversification of grasses. Nature. 2009;457(7229):551. 19189423 10.1038/nature07723

[23] Ohno S Other mechanisms for achieving gene duplication. Evolution by Gene Duplication. Springer; 1970 p. 107–10.

[24] Zhang HB, Zhao X, Ding X, Preparation of megabase‐size DNA from plant nuclei. Plant J. 1995;7(1):175–84.

[25] Langmead B, Salzberg SL Fast gapped-read alignment with Bowtie 2. Nat Methods. 2012;9(4):357–9. 22388286 10.1038/nmeth.1923 PMC3322381

[26] McKenna A, Hanna M, Banks E The Genome Analysis Toolkit: a MapReduce framework for analyzing next-generation DNA sequencing data. Genome Res. 2010;20(9):1297–303. 20644199 10.1101/gr.107524.110 PMC2928508

[27] Li H, Durbin R Fast and accurate short read alignment with Burrows–Wheeler transform. Bioinformatics. 2009;25(14):1754–60. 19451168 10.1093/bioinformatics/btp324 PMC2705234

[28] Bickhart DM, Rosen BD, Koren S, Single-molecule sequencing and chromatin conformation capture enable de novo reference assembly of the domestic goat genome. Nat Genet. 2017;49(4):643–50. 28263316 10.1038/ng.3802 PMC5909822

[29] Ou S, Jiang N LTR_retriever: a highly accurate and sensitive program for identification of long terminal-repeat retrotransposons. Plant Physiol. 2018;176:1410–22. 29233850 10.1104/pp.17.01310 PMC5813529

[30] Bolger AM, Lohse M, Usadel B Trimmomatic: a flexible trimmer for Illumina sequence data. Bioinformatics. 2014;30:2114–20. 24695404 10.1093/bioinformatics/btu170 PMC4103590

[31] Dobin A, Davis CA, Schlesinger F, STAR: ultrafast universal RNA-seq aligner. Bioinformatics. 2013;29(1):15–21. 23104886 10.1093/bioinformatics/bts635 PMC3530905

[32] Love MI, Huber W, Anders S Moderated estimation of fold change and dispersion for RNA-seq data with DESeq2. Genome Biol. 2014;15(12):550. 25516281 10.1186/s13059-014-0550-8 PMC4302049

[33] Kolde R Pheatmap: pretty heatmaps. R package version. 2012;61.

[34] Tang H, Wang X, Bowers JE, Unraveling ancient hexaploidy through multiply-aligned angiosperm gene maps. Genome Res. 2008;18(12):1944–54. 18832442 10.1101/gr.080978.108 PMC2593578

[35] MCscan GitHub wiki https://github.com/tanghaibao/jcvi/wiki/MCscan-(Python-version), GitHub, June 9th, 2018.

[36] VanBuren R, Wai CM, Colle M, Supporting data for “A near complete, chromosome-scale assembly of the black raspberry (Rubus occidentalis) genome”. GigaScience Database. 2018 10.5524/100465. PMC6131213 30107523

