# Supplementary material for: A near complete, chromosome-scale assembly of the black raspberry (Rubus occidentalis) genome
Source: Gigascience. 2018 Aug 9;7(8):giy094. doi: 10.1093/gigascience/giy094 (PMC6131213; doi:10.1093/gigascience/giy094)
Supplement: GIGA-D-18-00032_Original_Submission.pdf [file giy094_giga-d-18-00032_original_submission.pdf]

GigaScience

A near complete, chromosome-scale assembly of the black raspberry (Rubus occidentalis) genome

--Manuscript Draft--

**Manuscript Number:**	GIGA-D-18-00032
**Full Title:**	A near complete, chromosome-scale assembly of the black raspberry (Rubus occidentalis) genome
**Article Type:**	Research
**Funding Information:**	
**Abstract:**	Background<br>The fragmented nature of most draft plant genomes has hindered downstream gene discovery, trait mapping for breeding, and other functional genomics applications. There is a pressing need to improve or finish draft plant genome assemblies.<br><br>Findings<br>Here we present a chromosome-scale assembly of the black raspberry genome using single-molecule real-time (SMRT) PacBio sequencing and Hi-C genome scaffolding. The updated V3 assembly has a contig N50 of 5.1 Mb, representing a ~200-fold improvement over the previous Illumina-based version. Each of the 235 contigs was anchored and oriented into seven chromosomes, correcting several major misassemblies. Black raspberry V3 contains 47 Mb of new sequences including large pericentromeric regions and thousands of previously unannotated protein-coding genes. Among the new genes are hundreds of expanded tandem gene arrays that were collapsed in the Illumina-based assembly. Detailed comparative genomics with the high quality V4 woodland strawberry genome (Fragaria vesca) revealed near perfect 1:1 synteny with dramatic divergence in tandem gene array composition. Lineage-specific tandem gene arrays in black raspberry are related to agronomic traits such as disease resistance and secondary metabolite biosynthesis.<br><br>Conclusions<br>The improved resolution of tandem gene arrays highlights the need to reassemble these highly complex and biologically important regions in draft plant genomes. The updated, high-quality black raspberry reference genome will be useful for comparative genomics across the horticulturally important Rosaceae family and enable the development of marker assisted breeding in Rubus.
**Corresponding Author:**	Robert VanBuren<br><br>UNITED STATES
**Corresponding Author Secondary Information:**	
**Corresponding Author's Institution:**	
**Corresponding Author's Secondary Institution:**	
**First Author:**	Robert VanBuren
**First Author Secondary Information:**	
**Order of Authors:**	Robert VanBuren
	Ching Man Wai
	Marivi Colle
	Jie Wang
	Shawn Sullivan
	Jill M Bushakra

Powered by Editorial Manager® and ProduXion Manager® from Aries Systems Corporation

	Ivan Liachko
	Kelly Vining
	Michael Dossett
	Chad Finn
	David Chagne
	Rubina Jibran
	Kevin Childs
	Patrick P Edger
	Todd C Mockler
	Nahla V Bassil
**Order of Authors Secondary Information:**	
**Opposed Reviewers:**	
**Additional Information:**	
**Question**	**Response**
Are you submitting this manuscript to a special series or article collection?	No
**Experimental design and statistics**<br><br>Full details of the experimental design and statistical methods used should be given in the Methods section, as detailed in our Minimum Standards Reporting Checklist. Information essential to interpreting the data presented should be made available in the figure legends.<br><br>Have you included all the information requested in your manuscript?	Yes
**Resources**<br><br>A description of all resources used, including antibodies, cell lines, animals and software tools, with enough information to allow them to be uniquely identified, should be included in the Methods section. Authors are strongly encouraged to cite Research Resource Identifiers (RRIDs) for antibodies, model organisms and tools, where possible.<br><br>Have you included the information requested as detailed in our Minimum Standards Reporting Checklist?	Yes
**Availability of data and materials**	Yes

All datasets and code on which the conclusions of the paper rely must be either included in your submission or deposited in publicly available repositories (where available and ethically appropriate), referencing such data using a unique identifier in the references and in the "Availability of Data and Materials" section of your manuscript.<br><br>Have you have met the above requirement as detailed in our Minimum Standards Reporting Checklist?	

Powered by Editorial Manager® and ProduXion Manager® from Aries Systems Corporation

Manuscript

Click here to download Manuscript VanBuren_etal_Rubus_genome_MS_1-29-18.docx

A near complete, chromosome-scale assembly of the black raspberry (*Rubus occidentalis*) genome

Robert VanBuren[1,2]*, Ching Man Wai[1], Marivi Colle[1], Jie Wang[3], Shawn Sullivan[4], Jill M Bushakra[5], Ivan Liachko[4], Kelly J Vining[6], Michael Dossett[6], Chad E Finn[7], Rubina Jibran[8], David Chagné[8], Kevin Childs[3], Patrick P. Edger[1], Todd C. Mockler[9], Nahla V Bassil[5]

[1]Department of Horticulture, Michigan State University, East Lansing, MI, 48824, USA
[2]Plant Resilience Institute, Michigan State University, East Lansing, MI, 48824, USA
[3]Department of Plant Biology, Michigan State University, East Lansing, MI, 48824, USA
[4]Phase Genomics, Seattle, WA, 98195, USA
[5]USDA-ARS National Clonal Germplasm Repository, 33447 Peoria Rd., Corvallis, OR, 97333, USA
[6]Blueberry Council (in Partnership with Agriculture and Agri-Food Canada) Agassiz Food Research Centre, BC V0M 1A0, Canada
[7]USDA-ARS Horticultural Crops Research Unit, Corvallis, OR 97330, USA
[8]The New Zealand Institute for Plant & Food Research Limited, Private Bag 11600, Palmerston North 4474, New Zealand
[9]The Donald Danforth Plant Science Center, St. Louis, MO 63132, USA

* corresponding author: bobvanburen@gmail.com

Abstract

Background

The fragmented nature of most draft plant genomes has hindered downstream gene discovery, trait mapping for breeding, and other functional genomics applications. There is a pressing need to improve or finish draft plant genome assemblies.

Findings

Here we present a chromosome-scale assembly of the black raspberry genome using single-molecule real-time (SMRT) PacBio sequencing and Hi-C genome scaffolding. The updated V3 assembly has a contig N50 of 5.1 Mb, representing a ~200-fold improvement over the previous Illumina-based version. Each of the 235 contigs was anchored and oriented into seven chromosomes, correcting several major misassemblies. Black raspberry V3 contains 47 Mb of new sequences including large pericentromeric regions and thousands of previously unannotated protein-coding genes. Among the new genes are hundreds of expanded tandem gene arrays that were collapsed in the Illumina-based assembly. Detailed comparative genomics with the high quality V4 woodland strawberry genome (*Fragaria vesca*) revealed near perfect 1:1 synteny with dramatic divergence in tandem gene array composition. Lineage-specific tandem gene arrays in black raspberry are related to agronomic traits such as disease resistance and secondary metabolite biosynthesis.

Conclusions

The improved resolution of tandem gene arrays highlights the need to reassemble these highly complex and biologically important regions in draft plant genomes. The updated, high-quality black raspberry reference genome will be useful for comparative genomics across the horticulturally important Rosaceae family and enable the development of marker assisted breeding in *Rubus*.

Introduction

To date, over 200 plant genomes have been sequenced including most plants with agronomic value. Notable exceptions include large, polyploid, or otherwise complex genomes and many horticultural, medicinal or orphan crop species[1]. Most plant genomes were assembled using short read (50-500bp), next generation sequencing (NGS)-based approaches such as Illumina and 454 pyrosequencing technologies. The low cost and high-throughput of NGS technologies facilitated rapid genomic resource development, but the short read lengths produced low quality assemblies compared to the early Sanger-based plant genomes[1]. NGS-based assemblies contain gaps in repetitive regions that exceed the maximum read lengths, and most genomes have thousands to millions of imbedded sequence gaps. These gaps can span biologically important sequences including tandem gene arrays, repeat dense, and haplotype or homeologous specific regions. Recent advances in single molecule real-time sequencing (SMRT) have overcome the previous limitations of NGS-based approaches and ushered in a new era of 'platinum quality' reference genomes[2]. The long read lengths of PacBio- and Nanopore-based SMRT sequencing allow accurate assembly and phasing of complex genomic regions. SMRT sequencing has been used to drastically improve the contiguity of the maize[3], apple[4], woodland strawberry[5], and rice genomes[6] among others.

Black raspberry (*Rubus occidentalis* L.) is an important specialty fruit crop in the US Pacific Northwest that is closely related to the globally commercialized red raspberry (*R. idaeus* L.). Black raspberry has undergone little improvement since its domestication in the late 1800s[7] and elite cultivars suffer from limited genetic diversity[8, 9]. Genomic resources for *Rubus* are needed to accelerate marker assisted selection and improvement. The black raspberry genome was sequenced using an NGS-based approach, yielding a fragmented but much needed

draft assembly[10]. This draft was anchored into a chromosome scale assembly using a Hi-C-based scaffolding approach, but the reference used for scaffolding is ~ 50 Mb smaller than the estimated genome size, and is likely missing important genomic features. Here we utilized long read PacBio sequencing and Hi-C to finish and re-annotate the black raspberry genome. The updated V3 reference is nearly complete and includes thousands of new genes making it useful for the plant comparative genomics and *Rubus* breeding communities.

Results

To improve the black raspberry reference genome, we generated 2.1 million PacBio reads collectively spanning 21.8 Gb or 76x genome coverage. The PacBio data has a subread N50 length of 11.5 kb, average length of 9.8 kb, and maximum length of 72 kb (Supplemental Figure 1). PacBio reads shorter than 1 kb were discarded and reads longer than 10 kb were used as seeds for error correction and assembly using the Canu assembler [11]. The Canu-based assembly was improved by two rounds of polishing with Pilon [12] using high coverage (~80x) paired-end Illumina data to correct residual insertion/deletion errors. The final assembly has a contig N50 of 5.1 Mb across 235 contigs and total size of 290 Mb (Table 1). This represents a ~200x improvement in contiguity compared to the Illumina-only assembly and includes over 47 Mb of additional sequences. Newly assembled sequences consist of mostly repetitive elements but also include regions containing protein coding genes (described below). The Canu assembly graph is free of bubbles associated with heterozygous regions but there is some graph complexity resulting from high copy number repetitive elements (Figure 1b).

The PacBio-based contigs were assembled into scaffolds and then into pseudomolecules using high-throughput chromatin conformation capture (Hi-C) and Proximity-Guided Assembly (PGA). This approach was previously used to cluster and order 9,650 of the 11,936 V1 black raspberry contigs into seven pseudomolecules spanning 223.8 Mb (97.3% of the assembly). The Illumina-based Hi-C data was remapped to the PacBio assembly and clustered into seven pseudomolecules using the Proximo Hi-C scaffolding pipeline (Figure 1c). The Hi-C scaffolding was able to anchor and order with high confidence all 235 contigs into seven pseudomolecules with sizes ranging from 34 Mb to 51 Mb with an N50 of 41.1 Mb (Table 2). The pseudomolecules were assigned to the seven haploid black raspberry chromosomes (Ro01-Ro07) using markers from sequence-based genetic maps as anchors[13]. We used PBJelly[14] to fill gaps in the pseudomolecules with error-corrected PacBio reads exceeding 10 kb in length. This approach successfully filled 16 of the 228 gaps and the remaining gaps are likely either complex or highly repetitive with non-unique junctions exceeding read lengths.

The combined PacBio and Hi-C assembly (hereon referred to as V3) contains 10 terminal telomeric tracks at both ends of chromosomes Ro02, Ro03, Ro05, and Ro07, and one end of Ro01 and Ro04, validating the accuracy and quality of our assembly (Figure 2). We identified a novel 317 bp centromeric repeat with high abundance in six of the seven chromosomes. Centromeric repeat array sizes range from 110 elements in Ro01 to 1,204 in Ro04 with element homologies averaging 89% (Supplemental Table 1). The presence of centromeric arrays, repetitive element density, and Hi-C-based intra-chromosomal interactions allowed us to estimate the centromere size in each chromosome. Black raspberry chromosomes have an average centromere size of 2.8 Mb with individual sizes ranging from 173 kb in Ro01 to 5.2 Mb in Ro03. Ro06 contained only four centromeric repeats with no obvious enrichment of repetitive

elements or reduction in intra-chromosomal interactions based on the Hi-C data, suggesting the centromeric region of this chromosome is still largely unassembled. The proportion of long terminal repeat (LTR) retrotransposons in the black raspberry genome nearly doubled with an increase from 16.2% in V1 to 32.6% in V3. Intact LTR retrotransposons are a metric for assembly quality and the number of intact elements increased from 258 in V1 to 2,342 in V3. LTR and gene density are inversely correlated, with pericentromeric and subtelomeric regions having the highest LTR density (Figure 2). Together, the accurate assembly of highly repetitive regions and relatively low number of remaining sequence gaps suggest the V3 black raspberry assembly is nearly complete.

We aligned the V3 black raspberry assembly to the V1 pseudomolecules to assess genome collinearity. We identified numerous misassembles in V1 spanning most of the genome (Figure 1a). Misassembled regions range from small-scale inversions reflecting incorrect scaffold orientation, to major chromosome arm-sized inversions in Ro06 and Ro07. The pericentromeric regions are largely unassembled in V1 resulting in large gaps in the syntenic dot plots. Major gaps are also found throughout genic regions in the genome. The errors in V1 likely stem from read length limitations of NGS data and errors in marker order from the genetic maps that were used to build the pseudomolecules. A similar level of scaffold misassembly was observed in the comparison of PacBio based V4 woodland strawberry genome to the previous Illumina based genome[5]. Such errors are probably common in most NGS-based plant genomes and are hindering marker assisted breeding efforts.

The V3 black raspberry assembly includes 43 Mb of new sequences that was unassembled in the V1 reference. We re-annotated the V3 assembly *ab initio* using the MAKER-P pipeline [15]. Ten RNAseq datasets from a diverse tissue atlas were assembled with StringTie

[16] and used as transcript evidence and gene models from the diploid strawberry (*Fragaria vesca*) [5] and Arabidopsis (TAIR10) [17] genomes were used as protein evidence. The new annotation has 34,545 high-confidence gene models, substantially more than the 28,005 models in the V1 assembly. We assessed annotation quality using the Benchmarking Universal Single-Copy Orthologs (BUSCO) [18] pipeline and found 94% (1,352 out of 1,440) of the genes in the embryophyta dataset present in the V3 assembly, compared to 87% in the V1 black raspberry reference. This proportion is similar to other recent PacBio based genomes [2, 5, 19] and suggests the annotation is of high quality. The V3 annotation includes 9,301 new gene models that were improved or absent from the V1 assembly and 4,020 low-quality gene models from V1 were removed in V3. The discarded gene models had insufficient transcript or protein support, or transposable element related annotations. Most of the newly annotated genes (6,070 out of 9,301) have detectable expression in the gene expression atlas including many with tissue-specific expression patterns (Supplemental Figure 2).

The V3 black raspberry annotation has a striking increase in the size and number of tandem gene arrays. Tandem gene duplicates (TDs) with high sequence homology often collapse into single gene copies during the assembly of NGS data and are likely underrepresented in most genomes. We identified 7,453 TDs in the V3 assembly compared to 4,333 in V1. Tandem arrays range in size from 2 to 26 copies with an average size of 4. Large tandem arrays show the greatest improvement in assembly accuracy, with the most dramatic increase from four copies in V1 to 26 in V3 (Figure 3). Tandem arrays with more than 10 genes have, on average, 52% more annotated copies in V3. Tandem arrays with 5-9 genes have, on average, 31% more annotated copies in V3. Most arrays with 2 or 3 TDs are unchanged in the V3 assembly and 16% of arrays

were completely novel, with no homology to gene models in V1. Some differences in tandem array length are likely due to improvements in the annotation.

Black raspberry is in the Rosaceae, a large and diverse family that includes peach, pear, apple, strawberry, cherry, plum, rose, and almonds among other important horticultural crops. Genomes are available for many of these crop species providing an excellent framework for comparative functional genomic analyses. The closest crop relatives of black raspberry are the cultivated strawberries (*Fragaria* sp.), with the most common recent ancestor of these two species having diverged ~75 million years ago (MYA) [20]. Woodland strawberry (*F. vesca*) and black raspberry have the same karyotype (2*n*=14) and previous genetic map and genomic analyses suggested a high degree of collinearity[10]. We utilized the PacBio based V4 *F. vesca* assembly[5] to make detailed comparisons between these two species. Despite the 75 MY divergence, the black raspberry and *F. vesca* genomes are largely collinear (Figure 4). Ro01/Fvb1, Ro02/Fvb2, and Ro03/Fvb3 have no major structural rearrangements and the other four chromosome pairs have one or two major inversions (Figure 4a). Surprisingly, there are no translocations between chromosomes in either species. Over 96% of collinear blocks have 1:1 syntenic depth with no large-scale segmental duplications. The black raspberry and *F. vesca* genomes have 15,727 syntenic gene pairs which is consistent with other similarly diverged lineages such as species within the Poaceae [2, 21]. The black raspberry and *F. vesca* genomes are similar in size (290 vs. 240 Mb, respectively) and each genome has unique patterns of expansion based on microsynteny (Figure 4c). We identified 615 syntenic tandem gene arrays that are conserved between *F. vesca* and black raspberry, and 1,231 that are unique in either species. Syntenic TDs range in copy number, and no TDs with more than three copies have the same array size in both species. Most of the lineage-specific syntenic TDs have two or three

copies, but we identified 16 arrays with more than ten copies in black raspberry and only one copy in *F. vesca* (Supplementary Table 2). The most notable example is an expanded array of nucleotide-binding site leucine-rich repeat (NBS-LRR) proteins with 26 copies in black raspberry with only one copy in that gene in *F. vesca*. NBS-LRR proteins are involved in pathogen detection and are associated with many cloned disease resistance QTL [22].

The drastic improvements in the V3 black raspberry genome highlight the need to re-evaluate and improve most draft plant genomes. The cost of SMRT sequencing is continually decreasing, making it feasible to re-assemble even large and complex plant genomes. Most of the newly assembled black raspberry sequences are repetitive, but other collapsed regions such as tandem gene arrays were also drastically improved. Gene duplications drive evolutionary innovation [23] and these regions likely underlie important domestication and improvement related traits. Improving or finishing draft assemblies will help accelerate fundamental and applied plant research.

Methods:

DNA extraction and genome assembly

High molecular weight (HMW) genomic DNA (gDNA) was isolated from young leaf tissue of black raspberry selection ORUS 4115-3 using a modified nuclei preparation method[24]. A 20 kb insert library was constructed from the HMW gDNA followed by size selection on the BluePippin (Safe Science) and sequencing on a PacBio RSII platform using P6-C4 chemistry. Raw PacBio reads were corrected and assembled using the Canu assembler (V1.4)[11]. The following parameters were modified: minReadLength=2000, GenomeSize=290Mb,

minOverlapLength=1000. Other parameters were left as default. The PacBio-based contigs were polished with Pilon (V1.22) [12] using ~80x Illumina data from the V1 black raspberry draft genome assembly [10]. Quality-trimmed Illumina reads were aligned to the draft PacBio-based contigs using bowtie2 (V2.3.0)[25] with default parameters. The alignment rate of Illumina data was ~98%, supporting the completeness of our assembly. Illumina reads were realigned around insertions/deletions using the IndelRealigner function from the genome analysis tool kit (GATK; V3.7)[26]. The parameters for Pilon were as follows: --flank 7, --K 49, and --mindepth 20. Pilon was run a second time using the polished contigs as a reference to correct any residual errors. After two rounds of polishing, 431,421 indels and 95,322 single nucleotide polymorphisms were corrected in the assembly.

Pseudomolecule construction and validation

Hi-C library construction and sequencing was previously reported. In total, 54.4 million Hi-C read pairs were generated and used as input to the Proximo Hi-C scaffolding pipeline. Reads were aligned to the polished PacBio contigs using bwa (V0.7.16)[27] with strict parameters (-n 0) to prevent mismatches and non-specific alignments. Only read pairs that aligned to different contigs were used for scaffolding. The Proximo Hi-C pipeline performed chromosome clustering and contig orientation as described previously[28]. Briefly, Proximo utilizes an enhanced version of the LACHESIS algorithm as well as scaffold optimization and extra quality control steps to group and orient contigs based on interaction probabilities. Hi-C interactions binned the contigs into seven groups (corresponding to the haploid chromosomes) and successfully oriented all 235 contigs. Pseudomolecules were assigned to chromosomes using SSR and GBS based markers from high density genetic maps[13]. Gaps in the pseudomolecules were filled using error-

corrected PacBio reads with PBJelly (V 15.8.24)[14] using default parameters. This near complete version has been designated as V3.

Genome annotation

The MAKER-P pipeline[15] was used to annotate the V3 assembly. Ten RNAseq datasets (described below) used as transcript evidence and gene models from the diploid strawberry (*F. vesca*) [5] and Arabidopsis (TAIR10)[17] genomes were used as protein evidence. The RNAseq samples were assembled into transcripts using a reference-guided approach with StringTie (V1.3.3)[16]. A custom LTR retrotransposon library was created using the LTR_retriever pipeline[29]. This custom library was used in conjunction with the MAKER repeat library for masking prior to annotation. *Ab initio* gene prediction was performed using SNAP and Augustus with three and two rounds of reiterative training respectively. The resulting gene set was filtered to remove gene models containing Pfam domains related to transposable elements resulting in an annotation of 33,286 high-confidence gene models. 34,545 Annotation quality was assessed using the Benchmarking Universal Single-Copy Orthologs (BUSCO; V3)[18] pipeline with the embryophyta dataset of 1,440 single-copy conserved genes.

Expression analysis

To build a gene expression atlas, RNA was collected from ten diverse black raspberry tissues. This includes: green berries, red berries, ripe berries, flowers, canes, roots, leaves, and methyl jasmonate-treated leaf tissue. Fresh tissue was flash-frozen in liquid nitrogen and total RNA was extracted using KingFisher Pure RNA Plant kit (Thermo Fisher Scientific, MA), according to the

manufacturer's instructions. Two micrograms of total RNA was used to construct stranded mRNA libraries (KAPA mRNA HyperPrep kit, KAPA Biosystems, Roche, USA). Multiplexed, pooled libraries were sequenced on the Illumina HiSeq4000 under paired-end 150 nt mode in the genomics core at Michigan State University. Raw reads were trimmed using Trimmomatic (V 0.33)[30] and aligned to the black raspberry V3 genome using the STAR aligner[31]. Reads were then assembled using a reference-guided approach with StringTie (V1.3.3)[16] and output as read count tables. Expression analyses were performed using the DESeq2 pipeline[32] and visualized using the pheatmap R package[33].

Comparative genomics

The black raspberry V3 genome was compared to the black raspberry V1[10] and *F. vesca* V4[5] genomes using the MCScan toolkit (V1.1)[34]. Syntenic gene pairs were identified using all vs. all BLAST followed by filtering for 1:1 collinear pairs with MCScan. Tandem gene duplicates were identified using a minimum e-value of 1^{e-5} and maximum gene distance of 10 genes. Pair-wise, macrosynteny, and microsynteny plots were constructed using the python version of MCScan: (https://github.com/tanghaibao/jcvi/wiki/MCscan-(Python-version)).

Availability of supporting data: The updated black raspberry V3 assembly and annotation can be downloaded from CoGe (https://genomeevolution.org/coge) under Genome ID 37280 and the genome database for Rosaceae (https://www.rosaceae.org/). The raw sequence data have been deposited in the Short Read Archive (SRA) under NCBI BioProject ID PRJNA430858.

Competing Interests: The authors declare that they have no competing interests.

Author Contributions: R.V. and N.V.B. designed research; R.V., Je.W., M.C., Ji.W., S.S., J.M.B., I.L., K.J.V., M.D., C.E.F., R.J., D.C., K.C., P.P.E., T.C.M. and N.V.B. performed research and/or analyzed data; and R.V. wrote the paper. All authors reviewed the manuscript.

References:

1. Michael TP and VanBuren R. Progress, challenges and the future of crop genomes. Current opinion in plant biology. 2015;24:71-81.
2. VanBuren R, Bryant D, Edger PP, Tang H, Burgess D, Challabathula D, et al. Single-molecule sequencing of the desiccation-tolerant grass Oropetium thomaeum. Nature. 2015;527 7579:508-11.
3. Jiao Y, Peluso P, Shi J, Liang T, Stitzer MC, Wang B, et al. Improved maize reference genome with single-molecule technologies. Nature. 2017.
4. Daccord N, Celton J-M, Linsmith G, Becker C, Choisne N, Schijlen E, et al. High-quality de novo assembly of the apple genome and methylome dynamics of early fruit development. Nature Genetics. 2017.
5. Edger PP, VanBuren R, Colle M, Poorten TJ, Wai CM, Niederhuth CE, et al. Single-molecule sequencing and optical mapping yields an improved genome of woodland strawberry (Fragaria vesca) with chromosome-scale contiguity. GigaScience. 2017.
6. Du H, Yu Y, Ma Y, Gao Q, Cao Y, Chen Z, et al. Sequencing and de novo assembly of a near complete indica rice genome. Nature Communications. 2017;8:15324.
7. Jennings DL. Raspberries and blackberries: their breeding, diseases and growth. Academic press; 1988.
8. Dossett M, Bassil NV, Lewers KS and Finn CE. Genetic diversity in wild and cultivated black raspberry (Rubus occidentalis L.) evaluated by simple sequence repeat markers. Genetic resources and crop evolution. 2012;59 8:1849-65.
9. Dossett M, Lee J and Finn CE. Inheritance of phenological, vegetative, and fruit chemistry traits in black raspberry. Journal of the American Society for Horticultural Science. 2008;133 3:408-17.
10. VanBuren R, Bryant D, Bushakra JM, Vining KJ, Edger PP, Rowley ER, et al. The genome of black raspberry (Rubus occidentalis). The Plant Journal. 2016;87 6:535-47.
11. Koren S, Walenz BP, Berlin K, Miller JR, Bergman NH and Phillippy AM. Canu: scalable and accurate long-read assembly via adaptive k-mer weighting and repeat separation. bioRxiv. 2017:071282.
12. Walker BJ, Abeel T, Shea T, Priest M, Abouelliel A, Sakthikumar S, et al. Pilon: an integrated tool for comprehensive microbial variant detection and genome assembly improvement. PloS one. 2014;9 11:e112963.
13. Finn CE, Lee J, VanBuren R, Bassil NV, Bryant DW, Gilmore BS, et al. A genetic linkage map of black raspberry (Rubus occidentalis) and the mapping of Ag (4) conferring resistance to the aphid Amphorophora agathonica. 2015.
14. English AC, Richards S, Han Y, Wang M, Vee V, Qu J, et al. Mind the gap: upgrading genomes with Pacific Biosciences RS long-read sequencing technology. PloS one. 2012;7 11:e47768.
15. Campbell MS, Law M, Holt C, Stein JC, Moghe GD, Hufnagel DE, et al. MAKER-P: a tool kit for the rapid creation, management, and quality control of plant genome annotations. Plant physiology. 2014;164 2:513-24.
16. Pertea M, Pertea GM, Antonescu CM, Chang T-C, Mendell JT and Salzberg SL. StringTie enables improved reconstruction of a transcriptome from RNA-seq reads. Nature biotechnology. 2015;33 3:290-5.
17. Lamesch P, Berardini TZ, Li D, Swarbreck D, Wilks C, Sasidharan R, et al. The Arabidopsis Information Resource (TAIR): improved gene annotation and new tools. Nucleic acids research. 2011;40 D1:D1202-D10.

18. Simão FA, Waterhouse RM, Ioannidis P, Kriventseva EV and Zdobnov EM. BUSCO: assessing genome assembly and annotation completeness with single-copy orthologs. Bioinformatics. 2015;31 19:3210-2.
19. Jarvis DE, Ho YS, Lightfoot DJ, Schmöckel SM, Li B, Borm TJ, et al. The genome of Chenopodium quinoa. Nature. 2017;542 7641:307-12.
20. Xiang Y, Huang C-H, Hu Y, Wen J, Li S, Yi T, et al. Evolution of Rosaceae fruit types based on nuclear phylogeny in the context of geological times and genome duplication. Molecular biology and evolution. 2016;34 2:262-81.
21. Paterson AH, Bowers JE, Bruggmann R, Dubchak I, Grimwood J, Gundlach H, et al. The Sorghum bicolor genome and the diversification of grasses. Nature. 2009;457 7229:551-6.
22. Belkhadir Y, Subramaniam R and Dangl JL. Plant disease resistance protein signaling: NBS–LRR proteins and their partners. Current opinion in plant biology. 2004;7 4:391-9.
23. Ohno S. Other Mechanisms for Achieving Gene Duplication. Evolution by Gene Duplication. Springer; 1970. p. 107-10.
24. Zhang HB, Zhao X, Ding X, Paterson AH and Wing RA. Preparation of megabase-size DNA from plant nuclei. The Plant Journal. 1995;7 1:175-84.
25. Langmead B and Salzberg SL. Fast gapped-read alignment with Bowtie 2. Nature methods. 2012;9 4:357-9.
26. McKenna A, Hanna M, Banks E, Sivachenko A, Cibulskis K, Kernytsky A, et al. The Genome Analysis Toolkit: a MapReduce framework for analyzing next-generation DNA sequencing data. Genome research. 2010;20 9:1297-303.
27. Li H and Durbin R. Fast and accurate short read alignment with Burrows–Wheeler transform. Bioinformatics. 2009;25 14:1754-60.
28. Bickhart DM, Rosen BD, Koren S, Sayre BL, Hastie AR, Chan S, et al. Single-molecule sequencing and chromatin conformation capture enable de novo reference assembly of the domestic goat genome. Nature Genetics. 2017;49 4:643-50.
29. Ou S and Jiang N. LTR_retriever: a highly accurate and sensitive program for identification of long terminal-repeat retrotransposons. Plant Physiology. 2017:pp. 01310.2017.
30. Bolger AM, Lohse M and Usadel B. Trimmomatic: a flexible trimmer for Illumina sequence data. Bioinformatics. 2014:btu170.
31. Dobin A, Davis CA, Schlesinger F, Drenkow J, Zaleski C, Jha S, et al. STAR: ultrafast universal RNA-seq aligner. Bioinformatics. 2013;29 1:15-21.
32. Love MI, Huber W and Anders S. Moderated estimation of fold change and dispersion for RNA-seq data with DESeq2. Genome biology. 2014;15 12:550.
33. Kolde R. Pheatmap: pretty heatmaps. R package version. 2012;61.
34. Tang H, Wang X, Bowers JE, Ming R, Alam M and Paterson AH. Unraveling ancient hexaploidy through multiply-aligned angiosperm gene maps. Genome research. 2008;18 12:1944-54.

Figures:

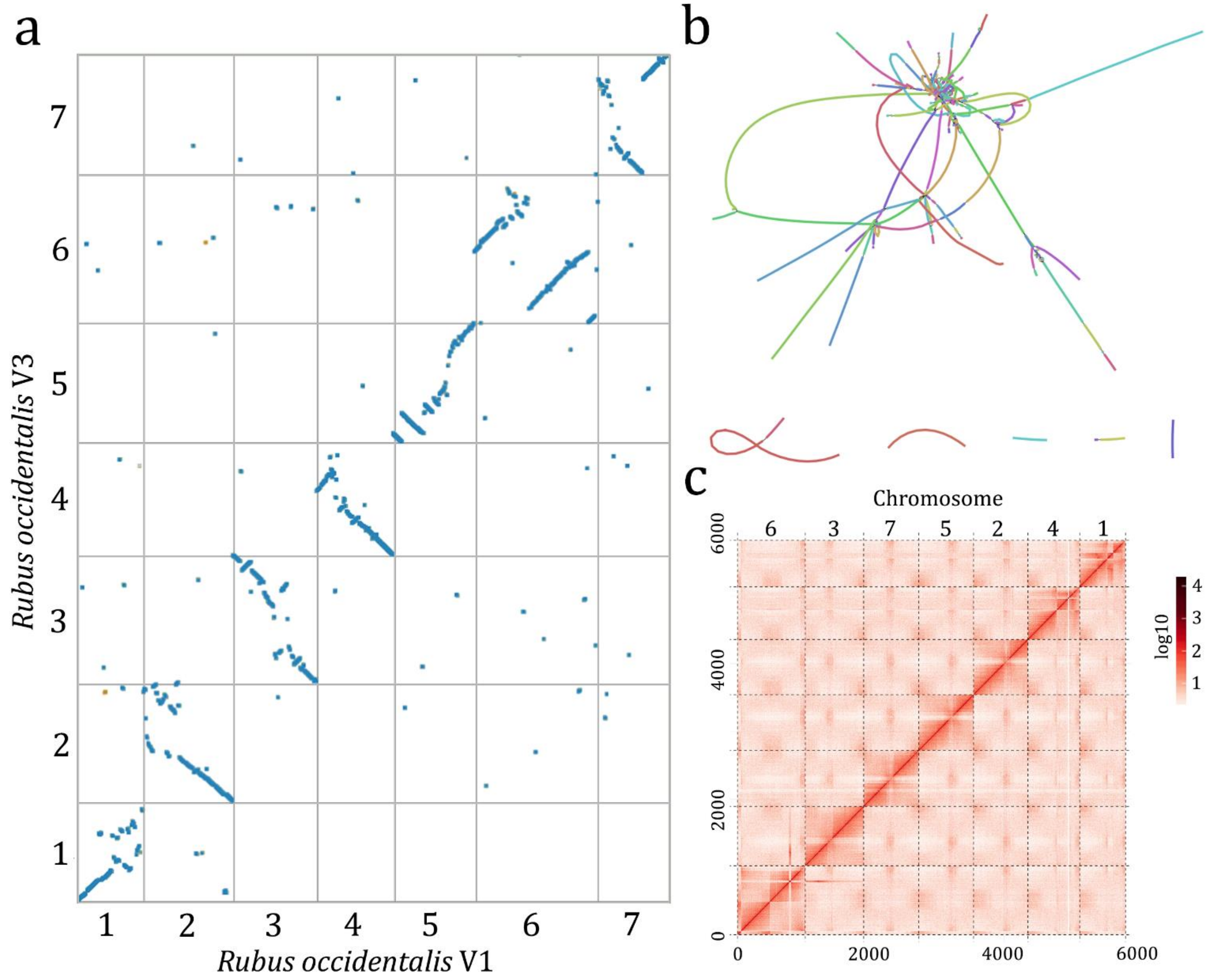

Figure 1. Updated chromosome scale assembly of black raspberry. (a) Syntenic dotplot of the black raspberry V1 and V3 assemblies. Each blue point denotes a collinear genomic region. (b) Assembly graph of the V3 reference. Each line (node) represents a contig in the Canu assembly and connections (edges) between contigs represent ambiguities in the graph structure. The color of contigs is randomly assigned. (c) Post-clustering heat map showing density of Hi-C interactions between contigs from the Proximity Guided Assembly.

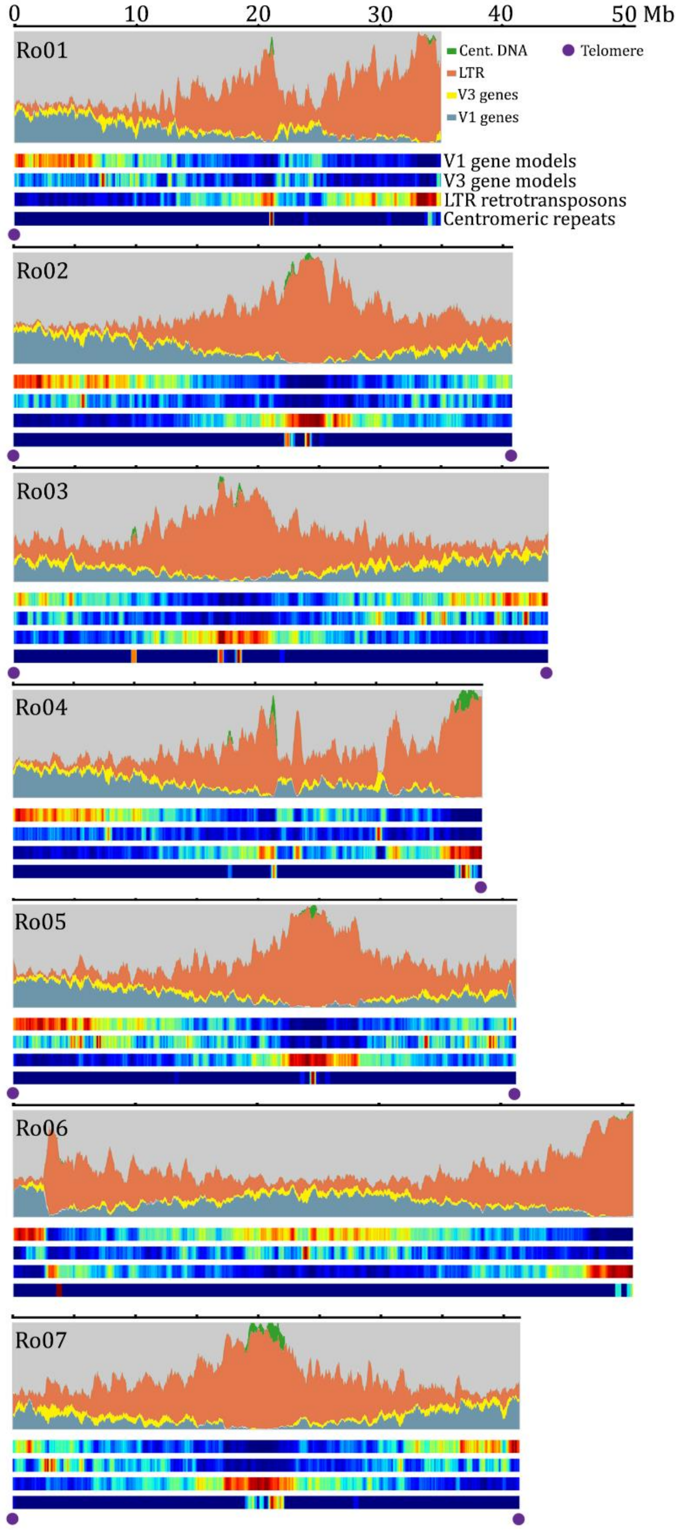

Figure 2: Genome landscape of the black raspberry V3 genome. The composition of long terminal repeat retrotransposons (LTRs), centromeric repeat arrays (Cent. DNA), gene models carried over from the V1 assembly, and new gene models in V3 are plotted in 50 kb bins with a 25kb sliding window. Terminal telomeric repeats are denoted by purple dots.

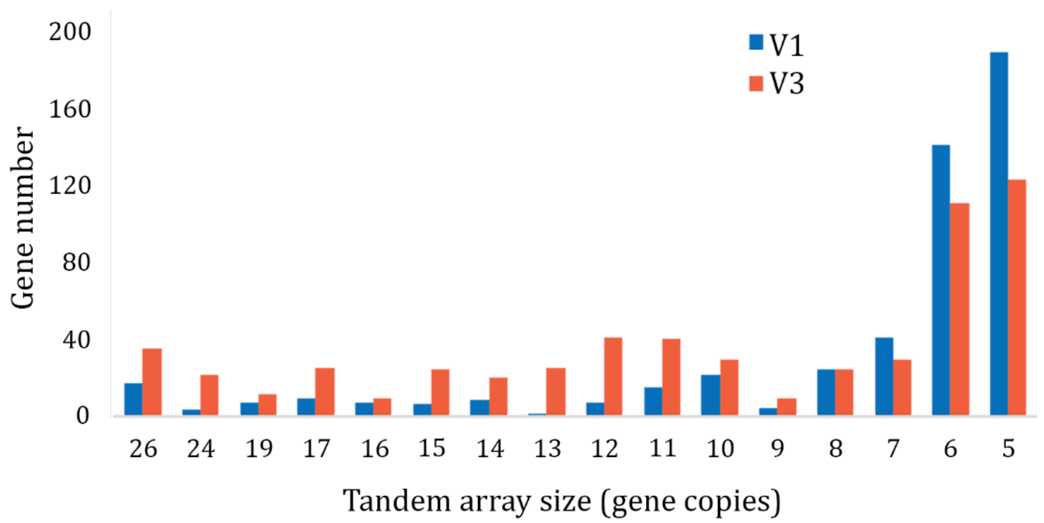

Figure 3. Comparison of tandem gene array sizes in the V1 and V3 black raspberry assemblies. The number of genes found in both the V1 and V3 assemblies (Blue) or only V3 (orange) is plotted for tandem arrays ranging in size from 5-26 copies. Array size is based on the V3 annotation.

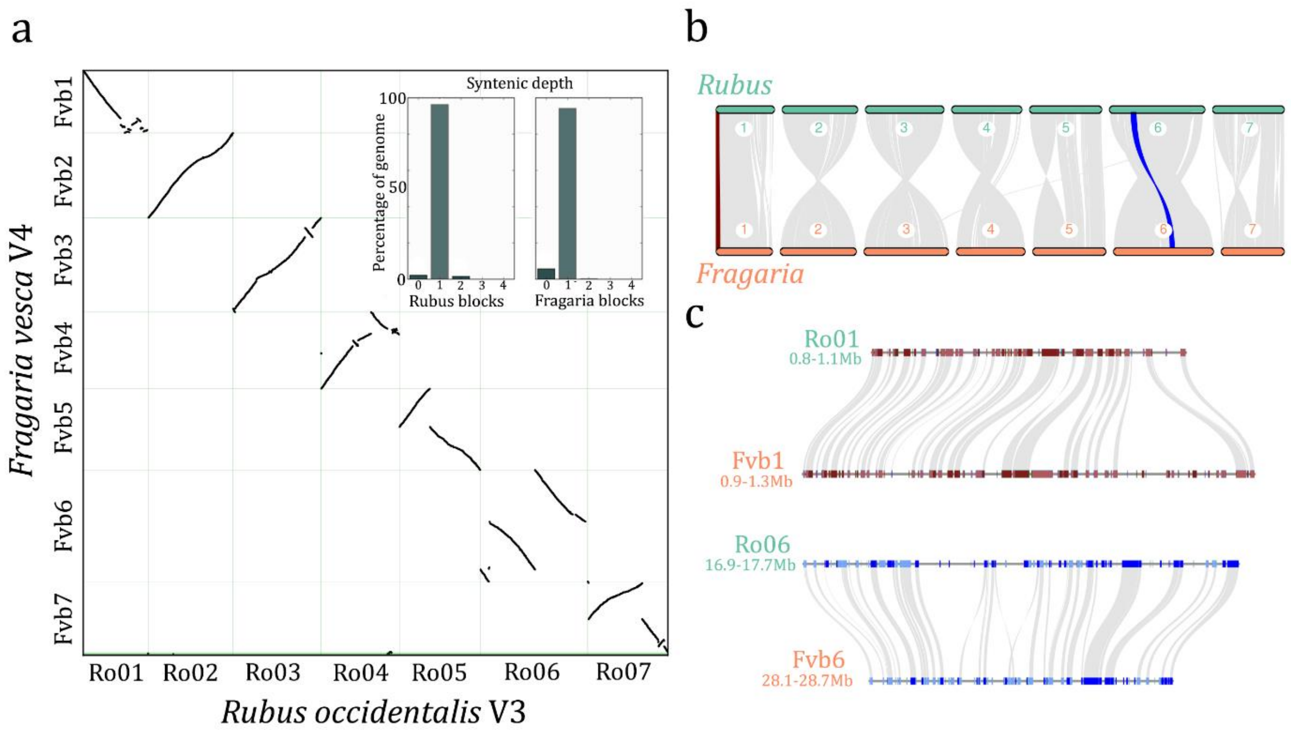

Figure 4. Comparative genomics of the black raspberry V3 and woodland strawberry (*Fragaria vesca*) V4 genomes. (a) Macrosyntenic dot plot between the black raspberry and *F. vesca* genomes. Each black dot represents a syntenic region between the two genomes. The inlaid bar graph shows syntenic depth of each red raspberry and *F. vesca* syntenic block. (b) Chromosome scale collinearity between black raspberry and *F. vesca*. The red collinear regions between Ro01 and Fvb1 blue regions between are Ro06 and Fvb6 shown in more detail in c. (c) Microsynteny of two regions showing lineage specific expansion in Fvb1 (top comparison) and Ro06 (bottom). Genes are shown in red or blue (top and bottom respectively) with colors indicating gene orientation (light are forward, dark are reverse). Syntenic gene pairs are connected by gray lines.

Table 1. Comparison of the black raspberry V1 and V3 assemblies

	V1	V3
**Number of contigs**	11,936	235
**Number of scaffolds**	2,226	7
**Contig N50**	33.1 kb	5.1 Mb
**Scaffold N50**	0.35 Mb	41.1 Mb
**LTR composition (%)**	16.20%	32.60%
**Number of genes**	28,005	34,545

Table 2. Summary of chromosome anchoring using the HiC genome map.

Chromosome	Anchored contigs	Total size (bp)
Ro01	19	34,302,027
Ro02	19	40,757,823
Ro03	30	43,767,452
Ro04	30	38,746,748
Ro05	25	41,095,993
Ro06	37	50,854,034
Ro07	75	41,277,220
**Total**	**235**	**290,801,297**

Click here to access/download
Supplementary Material
Supplement_1-19-18.docx
