# Supplementary material for: A near complete, chromosome-scale assembly of the black raspberry (Rubus occidentalis) genome
Source: Gigascience. 2018 Aug 9;7(8):giy094. doi: 10.1093/gigascience/giy094 (PMC6131213; doi:10.1093/gigascience/giy094)
Supplement: GIGA-D-18-00032_Revision_1.pdf [file giy094_giga-d-18-00032_revision_1.pdf]

GigaScience

A near complete, chromosome-scale assembly of the black raspberry (Rubus occidentalis) genome

--Manuscript Draft--

Manuscript Number:	GIGA-D-18-00032R1
Full Title:	A near complete, chromosome-scale assembly of the black raspberry (Rubus occidentalis) genome
Article Type:	Data Note
Funding Information:	
Abstract:	Background<br>The fragmented nature of most draft plant genomes has hindered downstream gene discovery, trait mapping for breeding, and other functional genomics applications. There is a pressing need to improve or finish draft plant genome assemblies.<br><br>Findings<br>Here we present a chromosome-scale assembly of the black raspberry genome using single-molecule real-time (SMRT) PacBio sequencing and Hi-C genome scaffolding. The updated V3 assembly has a contig N50 of 5.1 Mb, representing a ~200-fold improvement over the previous Illumina-based version. Each of the 235 contigs was anchored and oriented into seven chromosomes, correcting several major misassemblies. Black raspberry V3 contains 47 Mb of new sequences including large pericentromeric regions and thousands of previously unannotated protein-coding genes. Among the new genes are hundreds of expanded tandem gene arrays that were collapsed in the Illumina-based assembly. Detailed comparative genomics with the high quality V4 woodland strawberry genome (Fragaria vesca) revealed near perfect 1:1 synteny with dramatic divergence in tandem gene array composition. Lineage-specific tandem gene arrays in black raspberry are related to agronomic traits such as disease resistance and secondary metabolite biosynthesis.<br><br>Conclusions<br>The improved resolution of tandem gene arrays highlights the need to reassemble these highly complex and biologically important regions in draft plant genomes. The updated, high-quality black raspberry reference genome will be useful for comparative genomics across the horticulturally important Rosaceae family and enable the development of marker assisted breeding in Rubus.
Corresponding Author:	Robert VanBuren<br><br>UNITED STATES
Corresponding Author Secondary Information:	
Corresponding Author's Institution:	
Corresponding Author's Secondary Institution:	
First Author:	Robert VanBuren
First Author Secondary Information:	
Order of Authors:	Robert VanBuren
	Ching Man Wai
	Marivi Colle
	Jie Wang
	Shawn Sullivan
	Jill M Bushakra

Powered by Editorial Manager® and ProduXion Manager® from Aries Systems Corporation

	Ivan Liachko
	Kelly Vining
	Michael Dossett
	Chad Finn
	David Chagne
	Rubina Jibran
	Kevin Childs
	Patrick P Edger
	Todd C Mockler
	Nahla V Bassil
**Order of Authors Secondary Information:**	
**Response to Reviewers:**	Reviewer reports:<br>Reviewer #1: This manuscript presents a significantly improved genome assembly (and annotation) of the black raspberry (Rubus occidentalis). I am in line with the authors, that the new assembly will improve trait mapping for breeding, and other functional genomics applications, especially in regions of high complexity. However, I have several concerns that the authors should address prior to publication of the manuscript:<br><br>1) I consider the golden standard approach to polish any long read assembly to be a combination of long read polishing via Quiver or Arrow and short read polishing via Pilon. Authors only applied the latter, likely removing the majority of shorter mis-assemblies. However larger errors likely remained. The authors should add that their assembly might still contain larger erroneous regions which are not corrected by Pilon's short read approach.<br><br>R: In our personal experience, we have run into problems with misassemblies around tandem repeats and LTRs using Arrow, so we typically won’t use the PacBio data for error correction. HiC scaffolding failed to identify any misassembled contigs, supporting there are no large-scale errors still in the assembly. We agree with the reviewer that this could be a problem in our final assembly, and we have included discussion of possible misassemblies to the methods.<br><br>2) A detailed description of the methods used to define centromere sizes is missing. The authors should clearly lay out how the three lines of evidence, presence of centromeric arrays, repetitive element density, and Hi-C-based intra-chromosomal interactions were used to estimate the size of the centromere on each chromosome. Further they should investigate how much centromeric arrays / elements are in the unassembled reads that Canu usually returns, and speculate if their distribution mimics their assembled observation or could explain the missing centromeric repeats on Ro06.<br><br>R: It’s difficult to accurately define the borders of centromeres without cytological evidence, so the numbers reported here are rough estimates. Centromeres were identified using three lines of evidence: 1) reduced intrachromosomal interactions in the Hi-C heat map, 2) increased density of LTR retrotransposons, 3) presence of centromere specific tandem repeat arrays (317 bp). First, the intrachromosomal interactions were used to locate the putative centromere locations. Centromere locations were validated by overlap with centromere specific tandem repeat arrays. The estimated borders of centromeres were identified by the presence of centromere specific tandem repeats and LTR retrotransposon density > 85%. We have added this to the methods section.<br>The unassembled reads file from Canu contains 65,252 reads collectively spanning 569 Mb. Within the unassembled read file, only 9 reads (spanning 75kb) contain at least two copies of the centromeric repeat array. This suggests the centromeres are well assembled in black raspberry. These reads may correspond to the centromere in Ro06 or this centromere may contain no centromere specific repeat arrays.

3) BUSCO should not be used to assess whole "transcriptome" annotation quality per se but only to estimate completeness of a genome assembly. The authors should instead use http://hibberdlab.com/transrate to benchmark their annotation by re-mapping their RNA-seq reads to the old and the new reference, respectively the predicted transcripts.

R: We tried to run transrate (v1.0.3) but ran into several issues with the pipeline that are still unresolved based on discussion on GitHub. The transrate pipeline simply remaps RNAseq reads to the transcripts and references and outputs quality based metrics. This is a somewhat circular approach as the mapped RNAseq reads were used as transcript evidence for MAKER, so this approach could incorrectly inflate confidence in the annotation/assembly accuracy.
Roughly 96% of RNAseq reads map to the new assembly and the vast majority of these reads lie in predicted transcripts. Calculating the number of read mapping to transcripts is problematic as lncRNAs and TE are also expressed but were filtered from the annotation.

4) A definition of what the authors mean with tissue-specific expression pattern is missing. Both methods and results section should contain such a definition (including a definition of what is expressed and what is not). Results should be visualized using an intersection analysis, summarizing shared and tissue-specific expression patterns e.g., with a simple UpSetR plot.

R: Tissue specific expression is defined as having FPKM >1 in one tissue and FPKM < 1 for all other tissues. We have included this definition in the methods section. We have included an intersection analysis to summarize tissue specific and shared expression (see supplemental Figure 2).

5) The authors should add details about what the lineage specific expansion in Fvb1 (top comparison) and Ro06 (bottom) "contains". Is it linked 6) ?

R: These two panels represent two unrelated examples of regions with divergent gene composition between Rubus and Fragaria and are likely caused by a combination of lineage specific expansions/deletions in both genes and repetitive elements. We updated the text to discuss what these lineage specific regions likely contain (see lines 174-178):
“The black raspberry and F. vesca genomes are similar in size (290 vs. 240 Mb, respectively) and each genome has unique patterns of expansion/deletion based on gene-level microsynteny (Figure 4c). Differences in gene composition between species are likely due to a combination tandem gene duplications, retrotransposon mediated duplication/movement, fractionation/deletion, and mis-annotation. Expansion/deletion outside of genic regions is likely related to differences in repetitive element composition.”

6) The expanded NLR cluster is quite an interesting finding. Authors should add some more details about the "locus". Please classify the 26 genes according into TNL (TIR domain present), CNL (coiled-coiled domain present) or RNL (RPW8 domain present). Shortly elaborate on the differences between the 26 transcripts (if there are any) as well as on the expression of the transcripts. What is the tandem array status in the other Rosaceae? It would be further interesting to carry out a codeml analysis to test for lineage- and site-specific adaptive changes considering the F. vesca copy as outgroup (in case the single copy status is common in the Rosaceae).

R: We agree with the reviewer that the large array of NBS-LRR proteins is interesting, and we speculate that similarly large tandem arrays are misassembled in other genomes. These analyses are beyond the scope of this manuscript and we have removed this sentence for incorporation into future work.

8) The last paragraph, I consider it sort of a mix between discussion and summary, should be tailored towards future comparative analysis within the Rosaceae, how the new black raspberry genome can contribute or how it will help to "expedite the development of improved black and red raspberry, blackberry and other Rubus cultivars" (taken from the first genome paper) in detail. Please remove the "finishing

Powered by Editorial Manager® and ProduXion Manager® from Aries Systems Corporation

draft assemblies is important pitch" (everyone knew that when the first puzzly Illumina assemblies were released) and rewrite the whole paragraph.

R: We agree with the reviewer and have restructured the summary paragraph to better showcase the utility of the V3 black raspberry assembly.

Reviewer #2:

This new black raspberry assembly is a nice improvement upon an existing resource. Chromosome level assemblies are particularly useful in breeding efforts and comparative
genomics and are just easier to work with overall. The manuscript also provides some examples of genomic features that tend to be better assembled in PacBio assemblies versus
Illumina. The manuscript was well written, clear, and concise. I have a few comments:

1) For Pilon correction, what was the rationale for 2 rounds of correction? It may be be useful
to run more rounds until it reaches a plateau. Also, it wasn't clear if these were PE or SE
Illumina reads.

R: For most PacBio based genome projects, we usually run Pilon for 4-5 rounds or until the polishing plateaus. In this case, the first round of Pilon polishing corrected 102,366 Indels and 2,900 SNPs and 4,563 Indels and 0 SNPs were corrected in the second round. We attribute the ease of polishing to the high coverage of PacBio data, high coverage of PE Illumina data (~80x), and relatively simple genome structure. The high quality annotation and similarity to the Illumina based genome support the genome was adequately polished. We used PE ~300bp insert Illumina data from the Illumina based genome project for Pilon based polishing.

2) I was curious if other parameters for Canu or other assemblers, such as Falcon, were also
attempted in efforts to assemble the PacBio data. It can be good to run several assemblies
with the corrected reads to find the optimal one. If you have this data it would be nice to include it.

R: Given the relative simplicity of this genome (low heterozygosity, small size, and no recent LTR bursts), assembly using default parameters in Canu yielded a high quality assembly. This is supported by the relatively simple graph structure (see figure 1) and congruency with the HiC based anchoring. We did modify the following parameters for Canu: minReadLength=2000, GenomeSize=290Mb, minOverlapLength=1000. In more complex genomes, we will change the corrected output coverage, error rate, and overlapping options to phase or collapse haplotypes/repetitive regions. In our experience, Falcon produces similar assembly metrics to Canu, but is more likely to collapse repetitive regions. Because of this, we typically will not run Falcon.

3) What was the average size and range of sizes of gaps in the assembly? A supplemental
graph could show this.

R: The assembly contains 222 gaps across seven chromosomes after PBJelly based gap filling. Given the nature of HiC data, it is difficult to accurately estimate the physical size of gaps between anchored contigs. Optical maps and mate pair based scaffolding can be used to estimate gap size, but differences in chromatin interaction rates across the genome make this challenging with HiC data. All of the gaps were arbitrarily set to 100bp in this assembly. PBJelly was used to fill five of the gaps, but the remaining gaps are still 100 bp (though this is likely an underestimation). We have included gap lengths in the methods section.

4) It is stated that there were several misassemblies in the V1 genome compared to V3. How
do you know that none of these are errors in V3? Is it possible to use the existing

PacBio,
Illumina reads, or genetic maps to try to confirm this?

R: We agree with the reviewer and this is a great point. Identifying small-scale misassemblies in the Illumina based assembly is challenging given the fragmented nature of V1. We assembled ~47 Mb of new sequences and annotated thousands of new genes in V3, so there are numerous differences between assemblies. Large-scale errors in the V1 assembly are easier to identify. Maker density in the V1 map was relatively low and there were likely fine resolution marker order issues in this map stemming from erroneous marker calls from the GBS data. Because of this, there were likely ordering/orientation issues in the V1 pseudomolecules. We are currently remapping and analyzing the GBS data using the V3 reference, but this is beyond the scope of this manuscript. The HiC data was able to anchor and orient all 235 contigs with high confidence and no obvious misassemblies were identified. It is certainly possible there are misassemblies in V3, but they will be difficult to identify without additional lines of evidence. Similar assembly issues in Illumina based genomes have been identified in strawberry, apple, maize, and others.

5) What is meant by “high confidence” gene models? Did you use AED scores or something else to establish this?

R: High confidence was simply referring to models with AED scores < 1 that passed the default filtering thresholds by MAKER. We have removed the phrase ‘high confidence’ as we did not do any post filtering.

6) I think supplemental figure 2 could be included in the manuscript. If you do this, you should
have a scale to explain how the colors correspond to values and label the gene names in
panel b. I would also turn off clustering and keep the samples in the same order. Also, what
are you plotting here, FPKM, log CPM?

R: We have moved supplemental figure 2 to the main text and included a scale (log2 FPKM). B contains too many genes to include individual names. We chose to leave the clustering on to showcase that subsets of genes have tissue specific expression, which might explain why they were missed in the first annotation. Since the initial annotation is based on RNAseq data from only three tissues, many tissue specific genes were probably missed from a lack of transcript based evidence.

7) Please provide your Canu configuration file as a supplement and also the results of the
Augustus training (sensitivity/specificity). Did you provide your training set to Mario Stanke?

R: We listed the Canu parameters under the genome assembly section of the methods. We did not provide the training set to Mario Stanke.

8) I would like to see more comparison to the V1 annotation. How many genes overlap, how
many are new, how many were not found (you could make a Venn Diagram here). Also, are
the gene lengths comparable between the two annotations, or do you see longer gene lengths in your annotation due to the better assembly? Or are they shorter meaning some
residual indels may be breaking models?

R: We have added a Venn diagram to Figure 3 to show the annotation differences in V1 vs V3. In total, 25,244 gene models are shared between V1 and V3, 9,301 are new or greatly improved in V3, and 4,020 gene models from V1 were removed in V3. This is likely an artifact of improved assembly of gene space and an improved annotation pipeline.
The average gene length is roughly the same between both versions with an average gene length of 3,165 bp in V1 and 3,220 in V3. This suggests residual errors are

Powered by Editorial Manager® and ProduXion Manager® from Aries Systems Corporation

	probably not an issue in our new assembly.<br><br>9) Figure 2 would be clearer if the data was plotted on separate tracks. This could look nice as<br>a Circos figure.<br><br>R: We wanted to include all of the tracks on the same plot to make comparisons easier. For instance, overlaying gene and LTR density helps to clearly show the location of centromeric and pericentromeric regions. Circos plots are a good suggestion, but personally we find linear layouts of each chromosome easier to interpret.<br><br>10) For table one, please keep the same unit across the row for contig N50. Also, what is the<br>total assembly length and total repeat%?<br><br>R: We have updated Table 1 to include the total assembly length and standardized units. We annotated LTR transposons but did not annotate other repetitive elements, so we only included LTRs in this table.
**Additional Information:**	
**Question**	**Response**
Are you submitting this manuscript to a special series or article collection?	No
**Experimental design and statistics**<br><br>Full details of the experimental design and statistical methods used should be given in the Methods section, as detailed in our Minimum Standards Reporting Checklist. Information essential to interpreting the data presented should be made available in the figure legends.<br><br>Have you included all the information requested in your manuscript?	Yes
**Resources**<br><br>A description of all resources used, including antibodies, cell lines, animals and software tools, with enough information to allow them to be uniquely identified, should be included in the Methods section. Authors are strongly encouraged to cite Research Resource Identifiers (RRIDs) for antibodies, model organisms and tools, where possible.<br><br>Have you included the information requested as detailed in our Minimum	Yes

Powered by Editorial Manager® and ProduXion Manager® from Aries Systems Corporation

Standards Reporting Checklist?	
**Availability of data and materials**<br><br>All datasets and code on which the conclusions of the paper rely must be either included in your submission or deposited in publicly available repositories (where available and ethically appropriate), referencing such data using a unique identifier in the references and in the “Availability of Data and Materials” section of your manuscript.<br><br>Have you have met the above requirement as detailed in our Minimum Standards Reporting Checklist?	Yes

Manuscript
Click here to access/download;Manuscript;VanBuren_etal_Rubus_genome_M
Click here to view linked References

A near complete, chromosome-scale assembly of the black raspberry (*Rubus occidentalis*) genome

Robert VanBuren[1,2]*, Ching Man Wai[1], Marivi Colle[1], Jie Wang[3], Shawn Sullivan[4], Jill M Bushakra[5], Ivan Liachko[4], Kelly J Vining[6], Michael Dossett[6], Chad E Finn[7], Rubina Jibran[8], David Chagné[8], Kevin Childs[3], Patrick P. Edger[1], Todd C. Mockler[9], Nahla V Bassil[5]

[1]Department of Horticulture, Michigan State University, East Lansing, MI, 48824, USA
[2]Plant Resilience Institute, Michigan State University, East Lansing, MI, 48824, USA
[3]Department of Plant Biology, Michigan State University, East Lansing, MI, 48824, USA
[4]Phase Genomics, Seattle, WA, 98195, USA
[5]USDA-ARS National Clonal Germplasm Repository, 33447 Peoria Rd., Corvallis, OR, 97333, USA
[6]Blueberry Council (in Partnership with Agriculture and Agri-Food Canada) Agassiz Food Research Centre, BC V0M 1A0, Canada
[7]USDA-ARS Horticultural Crops Research Unit, Corvallis, OR 97330, USA
[8]The New Zealand Institute for Plant & Food Research Limited, Private Bag 11600, Palmerston North 4474, New Zealand
[9]The Donald Danforth Plant Science Center, St. Louis, MO 63132, USA

* corresponding author: bobvanburen@gmail.com

Abstract

Background

The fragmented nature of most draft plant genomes has hindered downstream gene discovery, trait mapping for breeding, and other functional genomics applications. There is a pressing need to improve or finish draft plant genome assemblies.

Findings

Here we present a chromosome-scale assembly of the black raspberry genome using single-molecule real-time (SMRT) PacBio sequencing and Hi-C genome scaffolding. The updated V3 assembly has a contig N50 of 5.1 Mb, representing a ~200-fold improvement over the previous Illumina-based version. Each of the 235 contigs was anchored and oriented into seven chromosomes, correcting several major misassemblies. Black raspberry V3 contains 47 Mb of new sequences including large pericentromeric regions and thousands of previously unannotated protein-coding genes. Among the new genes are hundreds of expanded tandem gene arrays that were collapsed in the Illumina-based assembly. Detailed comparative genomics with the high quality V4 woodland strawberry genome (*Fragaria vesca*) revealed near perfect 1:1 synteny with dramatic divergence in tandem gene array composition. Lineage-specific tandem gene arrays in black raspberry are related to agronomic traits such as disease resistance and secondary metabolite biosynthesis.

Conclusions

The improved resolution of tandem gene arrays highlights the need to reassemble these highly complex and biologically important regions in draft plant genomes. The updated, high-quality black raspberry reference genome will be useful for comparative genomics across the horticulturally important Rosaceae family and enable the development of marker assisted breeding in *Rubus*.

Introduction

To date, over 200 plant genomes have been sequenced including most plants with agronomic value. Notable exceptions include large, polyploid, or otherwise complex genomes and many horticultural, medicinal or orphan crop species[1]. Most plant genomes were assembled using short read (50-500bp), next generation sequencing (NGS)-based approaches such as Illumina and 454 pyrosequencing technologies. The low cost and high-throughput of NGS technologies facilitated rapid genomic resource development, but the short read lengths produced low quality assemblies compared to the early Sanger-based plant genomes[1]. NGS-based assemblies contain gaps in repetitive regions that exceed the maximum read lengths, and most genomes have thousands to millions of imbedded sequence gaps. These gaps can span biologically important sequences including tandem gene arrays, repeat dense, and haplotype or homeologous specific regions. Recent advances in single molecule real-time sequencing (SMRT) have overcome the previous limitations of NGS-based approaches and ushered in a new era of 'platinum quality' reference genomes[2]. The long read lengths of PacBio- and Nanopore-based SMRT sequencing allow accurate assembly and phasing of complex genomic regions. SMRT sequencing has been used to drastically improve the contiguity of the maize[3], apple[4], woodland strawberry[5], and rice genomes[6] among others.

Black raspberry (*Rubus occidentalis* L.) is an important specialty fruit crop in the US Pacific Northwest that is closely related to the globally commercialized red raspberry (*R. idaeus* L.). Black raspberry has undergone little improvement since its domestication in the late 1800s[7] and elite cultivars suffer from limited genetic diversity[8, 9]. Genomic resources for *Rubus* are needed to accelerate marker assisted selection and improvement. The black raspberry genome was sequenced using an NGS-based approach, yielding a fragmented but much needed

draft assembly[10]. This draft was anchored into a chromosome scale assembly using a Hi-C-based scaffolding approach [11], but the reference used for scaffolding is ~ 50 Mb smaller than the estimated genome size, and is likely missing important genomic features. Here we utilized long read PacBio sequencing and Hi-C to finish and re-annotate the black raspberry genome. The updated V3 reference is nearly complete and includes thousands of new genes making it useful for the plant comparative genomics and *Rubus* breeding communities.

Results

To improve the black raspberry reference genome, we generated 2.1 million PacBio reads collectively spanning 21.8 Gb or 76x genome coverage. The PacBio data has a subread N50 length of 11.5 kb, average length of 9.8 kb, and maximum length of 72 kb (Supplemental Figure 1). PacBio reads shorter than 1 kb were discarded and reads longer than 10 kb were used as seeds for error correction and assembly using the Canu assembler [12]. The Canu-based assembly was improved by two rounds of polishing with Pilon [13] using high coverage (~80x) paired-end Illumina data to correct residual insertion/deletion errors. The final assembly has a contig N50 of 5.1 Mb across 235 contigs and total size of 290 Mb (Table 1). This represents a ~200x improvement in contiguity compared to the Illumina-only assembly and includes over 47 Mb of additional sequences. Newly assembled sequences consist of mostly repetitive elements but also include regions containing protein coding genes (described below). The Canu assembly graph is free of bubbles associated with heterozygous regions but there is some graph complexity resulting from high copy number repetitive elements (Figure 1b).

The PacBio-based contigs were assembled into scaffolds and then into pseudomolecules using high-throughput chromatin conformation capture (Hi-C) and Proximity-Guided Assembly (PGA). This approach was previously used by Jibran et al. [11] to cluster and order 9,650 of the 11,936 V1 black raspberry contigs into seven pseudomolecules spanning 223.8 Mb (97.3% of the assembly. The Illumina-based Hi-C data from Jibran et al. [11] was remapped to the PacBio assembly and clustered into seven pseudomolecules using the Proximo Hi-C scaffolding pipeline (Figure 1c). The Hi-C scaffolding was able to anchor and order with high confidence all 235 contigs into seven pseudomolecules with sizes ranging from 34 Mb to 51 Mb with an N50 of 41.1 Mb (Table 2). The pseudomolecules were assigned to the seven haploid black raspberry chromosomes (Ro01-Ro07) using markers from sequence-based genetic maps as anchors[14]. We used PBJelly [15] to fill gaps in the pseudomolecules with error-corrected PacBio reads exceeding 10 kb in length. This approach successfully filled 16 of the 228 gaps and the remaining gaps are likely either complex or highly repetitive with non-unique junctions exceeding read lengths.

The combined PacBio and Hi-C assembly (hereon referred to as V3) contains 10 terminal telomeric tracks at both ends of chromosomes Ro02, Ro03, Ro05, and Ro07, and one end of Ro01 and Ro04, validating the accuracy and quality of our assembly (Figure 2). We identified a novel 317 bp centromeric repeat with high abundance in six of the seven chromosomes. Centromeric repeat array sizes range from 110 elements in Ro01 to 1,204 in Ro04 with element homologies averaging 89% (Supplemental Table 1). The presence of centromeric arrays, repetitive element density, and Hi-C-based intra-chromosomal interactions allowed us to estimate the centromere size in each chromosome. Black raspberry chromosomes have an average centromere size of 2.8 Mb with individual sizes ranging from 173 kb in Ro01 to 5.2 Mb

in Ro03. Ro06 contained only four centromeric repeats with no obvious enrichment of repetitive elements or reduction in intra-chromosomal interactions based on the Hi-C data, suggesting the centromeric region of this chromosome is still largely unassembled. The proportion of long terminal repeat (LTR) retrotransposons in the black raspberry genome nearly doubled with an increase from 16.2% in V1 to 32.6% in V3. Intact LTR retrotransposons are a metric for assembly quality and the number of intact elements increased from 258 in V1 to 2,342 in V3. LTR and gene density are inversely correlated, with pericentromeric and subtelomeric regions having the highest LTR density (Figure 2). Together, the accurate assembly of highly repetitive regions and relatively low number of remaining sequence gaps suggest the V3 black raspberry assembly is nearly complete.

We aligned the V3 black raspberry assembly to the V1 pseudomolecules to assess genome collinearity. We identified numerous misassembles in V1 spanning most of the genome (Figure 1a). Misassembled regions range from small-scale inversions reflecting incorrect scaffold orientation, to major chromosome arm-sized inversions in Ro06 and Ro07. The pericentromeric regions are largely unassembled in V1 resulting in large gaps in the syntenic dot plots. Major gaps are also found throughout genic regions in the genome. The errors in V1 likely stem from read length limitations of NGS data and errors in marker order from the genetic maps that were used to build the pseudomolecules. A similar level of scaffold misassembly was observed in the comparison of PacBio based V4 woodland strawberry genome to the previous Illumina based genome[5]. Such errors are probably common in most NGS-based plant genomes and are hindering marker assisted breeding efforts.

The V3 black raspberry assembly includes 43 Mb of new sequences that was unassembled in the V1 reference. We re-annotated the V3 assembly *ab initio* using the MAKER-

P pipeline [16]. Ten RNAseq datasets from a diverse tissue atlas were assembled with StringTie [17] and used as transcript evidence and gene models from the diploid strawberry (*Fragaria vesca*) [5] and Arabidopsis (TAIR10) [18] genomes were used as protein evidence. The new annotation has 34,545 high-confidence gene models, substantially more than the 28,005 models in the V1 assembly. We assessed annotation quality using the Benchmarking Universal Single-Copy Orthologs [19] pipeline and found 94% (1,352 out of 1,440) of the genes in the embryophyta dataset present in the V3 assembly, compared to 87% in the V1 black raspberry reference. This proportion is similar to other recent PacBio based genomes [5, 20, 21] and suggests the annotation is of high quality. The V3 annotation includes 9,301 new gene models that were improved or absent from the V1 assembly and 4,020 low-quality gene models from V1 were removed in V3. The discarded gene models had insufficient transcript or protein support, or transposable element related annotations. The average gene length is 3,165 bp and 3,220 bp for V1 and V3 respectively, suggesting residual indels in V3 are not resulting in fragmented gene models. Most of the newly annotated genes (6,070 out of 9,301) have detectable expression (FPKM > 1) in the gene expression atlas (Figure 4). Many new genes have tissue-specific expression patterns (Supplemental Figure 2), which may explain why they were missed in the V1 annotation.

The V3 black raspberry annotation has a striking increase in the size and number of tandem gene arrays. Tandem gene duplicates (TDs) with high sequence homology often collapse into single gene copies during the assembly of NGS data and are likely underrepresented in most genomes. We identified 7,453 TDs in the V3 assembly compared to 4,333 in V1. Tandem arrays range in size from 2 to 26 copies with an average size of 4. Large tandem arrays show the greatest improvement in assembly accuracy, with the most dramatic increase from four copies in

V1 to 26 in V3 (Figure 3). Tandem arrays with more than 10 genes have, on average, 52% more annotated copies in V3. Tandem arrays with 5-9 genes have, on average, 31% more annotated copies in V3. Most arrays with 2 or 3 TDs are unchanged in the V3 assembly and 16% of arrays were completely novel, with no homology to gene models in V1. Some differences in tandem array length are likely due to improvements in the annotation.

Black raspberry is in the Rosaceae, a large and diverse family that includes peach, pear, apple, strawberry, cherry, plum, rose, and almonds among other important horticultural crops. Genomes are available for many of these crop species providing an excellent framework for comparative functional genomic analyses. The closest crop relatives of black raspberry are the cultivated strawberries (*Fragaria* sp.), with the most common recent ancestor of these two species having diverged ~75 million years ago (MYA) [22]. Woodland strawberry (*F. vesca*) and black raspberry have the same karyotype ($2n$=14) and previous genetic map and genomic analyses suggested a high degree of collinearity[10]. We utilized the PacBio based V4 *F. vesca* assembly[5] to make detailed comparisons between these two species. Despite the 75 MY divergence, the black raspberry and *F. vesca* genomes are largely collinear (Figure 5). Ro01/Fvb1, Ro02/Fvb2, and Ro03/Fvb3 have no major structural rearrangements and the other four chromosome pairs have one or two major inversions (Figure 5a). Surprisingly, there are no translocations between chromosomes in either species. Over 96% of collinear blocks have 1:1 syntenic depth with no large-scale segmental duplications. The black raspberry and *F. vesca* genomes have 15,727 syntenic gene pairs which is consistent with other similarly diverged lineages such as species within Poaceae [2, 23]. The black raspberry and *F. vesca* genomes are similar in size (290 vs. 240 Mb, respectively) and each genome has unique patterns of expansion/deletion based on gene-level microsynteny (Figure 5c). Differences in gene

composition between species are likely due to a combination tandem gene duplications, retrotransposon mediated duplication/movement, fractionation/deletion, and mis-annotation. Expansion/deletion outside of genic regions is likely related to differences in repetitive element composition. We identified 615 syntenic tandem gene arrays that are conserved between *F. vesca* and black raspberry, and 1,231 that are unique in either species. Syntenic TDs range in copy number, and no TDs with more than three copies have the same array size in both species. Most of the lineage-specific syntenic TDs have two or three copies, but we identified 16 arrays with more than ten copies in black raspberry and only one copy in *F. vesca* (Supplementary Table 2).

The drastic improvements in the V3 black raspberry genome will help accelerate marker assisted selection and functional genomics studies in *Rubus*. Most of the newly assembled black raspberry sequences are repetitive, but other collapsed regions such as tandem gene arrays were also drastically improved. Gene duplications drive evolutionary innovation [24] and these regions likely underlie important domestication and improvement related traits. The black raspberry gene expression atlas also helped improve the structure of several thousand existing and new gene models that were incorrectly annotated or missing due to a lack of transcript support. Numerous scaffolding errors were fixed in V3 and the corrected pseudomolecules will facilitate more accurate marker ordering and fine mapping for QTL and GWAS studies. Black raspberry joins a growing list of near-complete, reference grade genome assemblies for the plant comparative genomics community.

Methods:

DNA extraction and genome assembly

High molecular weight (HMW) genomic DNA (gDNA) was isolated from young leaf tissue of black raspberry selection ORUS 4115-3 using a modified nuclei preparation method[25]. A 20 kb insert library was constructed from the HMW gDNA followed by size selection on the BluePippin (Safe Science) and sequencing on a PacBio RSII platform using P6-C4 chemistry. Raw PacBio reads were corrected and assembled using the Canu assembler V1.4 (Canu, RRID:SCR_015880) [12]. The following parameters were modified: minReadLength=2000, GenomeSize=290Mb, minOverlapLength=1000. Other parameters were left as default. The PacBio-based contigs were polished with Pilon V1.22 (Pilon, RRID:SCR_014731) [13] using ~80x Illumina PE data from the V1 black raspberry draft genome assembly [10]. Quality-trimmed Illumina reads were aligned to the draft PacBio-based contigs using bowtie2 (V2.3.0)[26] with default parameters. The alignment rate of Illumina data was ~98%, supporting the completeness of our assembly. Illumina reads were realigned around insertions/deletions using the IndelRealigner function from the genome analysis tool kit V3.7 (GATK, RRID:SCR_001876), [27]. The parameters for Pilon were as follows: --flank 7, --K 49, and --mindepth 20. Pilon was run a second time using the polished contigs as a reference to correct any residual errors. After two rounds of polishing, 106,929 indels and 2,900 single nucleotide polymorphisms were corrected in the assembly. Since the assembly was not polished using the PacBio reads, it is possible long range misassembles were missed by Pilon and may still be present in our assembly.

Pseudomolecule construction and validation

Hi-C library construction and sequencing was previously reported [11]. In total, 54.4 million Hi-C read pairs from Jibran et al. [11] were generated and used as input to the Proximo Hi-C

scaffolding pipeline. Reads were aligned to the polished PacBio contigs using BWA V0.7.16 (BWA, RRID:SCR_010910) [28] with strict parameters (-n 0) to prevent mismatches and non-specific alignments. Only read pairs that aligned to different contigs were used for scaffolding. The Proximo Hi-C pipeline performed chromosome clustering and contig orientation as described previously[29]. Briefly, Proximo utilizes an enhanced version of the LACHESIS algorithm as well as scaffold optimization and extra quality control steps to group and orient contigs based on interaction probabilities. Hi-C interactions binned the contigs into seven groups (corresponding to the haploid chromosomes) and successfully oriented all 235 contigs. The gap length between ordered contigs was set at 100 bp. Pseudomolecules were assigned to chromosomes using SSR and GBS based markers from high density genetic maps[14]. Gaps in the pseudomolecules were filled using error-corrected PacBio reads with PBJelly V 15.8.24 (PBJelly, RRID:SCR_012091)[15] using default parameters. This near complete version has been designated as V3.

Identification of centromeric regions

Centromeres were identified using three lines of evidence: 1) reduced intrachromosomal interactions in the Hi-C heat map, 2) increased density of LTR retrotransposons, 3) presence of centromere specific tandem repeat arrays (317 bp). First, the intrachromosomal interactions were used to locate the putative centromere locations. Centromere locations were validated by overlap with centromere specific tandem repeat arrays. The estimated borders of centromeres were identified by the presence of centromere specific tandem repeats and LTR retrotransposon density > 85%. Putative centromeres were identified for six of the seven black raspberry chromosomes, and no enrichment of centromere tandem repeats was found in Ro06. Centromere

specific repeat arrays were found in only nine reads in the unassembled read file from Canu, suggesting the centromeres are well assembled in black raspberry.

Genome annotation

The MAKER-P pipeline (MAKER, RRID:SCR_005309)[16] was used to annotate the V3 assembly. Ten RNAseq datasets (described below) used as transcript evidence and gene models from the diploid strawberry (*F. vesca*) [5] and Arabidopsis (TAIR10)[18] genomes were used as protein evidence. The RNAseq samples were assembled into transcripts using a reference-guided approach with StringTie (V1.3.3)[17]. A custom LTR retrotransposon library was created using the LTR_retriever pipeline[30]. This custom library was used in conjunction with the MAKER repeat library for masking prior to annotation. *Ab initio* gene prediction was performed using SNAP and Augustus (Augustus: Gene Prediction, RRID:SCR_008417) with three and two rounds of reiterative training respectively. The resulting gene set was filtered to remove gene models containing Pfam domains related to transposable elements resulting in an annotation of 33,286 gene models. Annotation quality was assessed using the Benchmarking Universal Single-Copy Orthologs V3 (BUSCO, RRID:SCR_015008)[19] pipeline with the embryophyta dataset of 1,440 single-copy conserved genes.

Expression analysis

To build a gene expression atlas, RNA was collected from ten diverse black raspberry tissues. This includes: green berries, red berries, ripe berries, flowers, canes, roots, leaves, and methyl jasmonate-treated leaf tissue. Fresh tissue was flash-frozen in liquid nitrogen and total RNA was extracted using KingFisher Pure RNA Plant kit (Thermo Fisher Scientific, MA), according to the

manufacturer's instructions. Two micrograms of total RNA was used to construct stranded mRNA libraries (KAPA mRNA HyperPrep kit, KAPA Biosystems, Roche, USA). Multiplexed, pooled libraries were sequenced on the Illumina HiSeq4000 under paired-end 150 nt mode in the genomics core at Michigan State University. Raw reads were trimmed using Trimmomatic V 0.33 (Trimmomatic, RRID:SCR_011848) [31] and aligned to the black raspberry V3 genome using the STAR aligner[32]. Reads were then assembled using a reference-guided approach with StringTie (V1.3.3)[17] and output as read count tables. Expression analyses were performed using the DESeq2 pipeline[33] and visualized using the pheatmap R package[34]. Tissue specific expression was defined as having > 1 FPKM in one tissue and FPKM < 1 in all other tissues.

Comparative genomics

The black raspberry V3 genome was compared to the black raspberry V1[10] and *F. vesca* V4[5] genomes using the MCScan toolkit (V1.1)[35]. Syntenic gene pairs were identified using all vs. all BLAST followed by filtering for 1:1 collinear pairs with MCScan. Tandem gene duplicates were identified using a minimum e-value of 1^{e-5} and maximum gene distance of 10 genes. Pair-wise, macrosynteny, and microsynteny plots were constructed using the python version of MCScan: (https://github.com/tanghaibao/jcvi/wiki/MCscan-(Python-version)).

Availability of supporting data: The updated black raspberry V3 assembly and annotation can be downloaded from CoGe (https://genomeevolution.org/coge) under Genome ID 37280 and the genome database for Rosaceae (https://www.rosaceae.org/). The raw sequence data have been

deposited in the Short Read Archive (SRA) under NCBI BioProject ID PRJNA430858. Supporting data are also available via the GigaDB dataset for this project [36].

Competing Interests: The authors declare that they have no competing interests.

Author Contributions: R.V. and N.V.B. designed research; R.V., Je.W., M.C., Ji.W., S.S., J.M.B., I.L., K.J.V., M.D., C.E.F., R.J., D.C., K.C., P.P.E., T.C.M. and N.V.B. performed research and/or analyzed data; and R.V. wrote the paper. All authors reviewed the manuscript.

References:

1. Michael TP and VanBuren R. Progress, challenges and the future of crop genomes. Current opinion in plant biology. 2015;24:71-81.
2. VanBuren R, Bryant D, Edger PP, Tang H, Burgess D, Challabathula D, et al. Single-molecule sequencing of the desiccation-tolerant grass Oropetium thomaeum. Nature. 2015;527 7579:508-11.
3. Jiao Y, Peluso P, Shi J, Liang T, Stitzer MC, Wang B, et al. Improved maize reference genome with single-molecule technologies. Nature. 2017.
4. Daccord N, Celton J-M, Linsmith G, Becker C, Choisne N, Schijlen E, et al. High-quality de novo assembly of the apple genome and methylome dynamics of early fruit development. Nature Genetics. 2017.
5. Edger PP, VanBuren R, Colle M, Poorten TJ, Wai CM, Niederhuth CE, et al. Single-molecule sequencing and optical mapping yields an improved genome of woodland strawberry (Fragaria vesca) with chromosome-scale contiguity. GigaScience. 2017.
6. Du H, Yu Y, Ma Y, Gao Q, Cao Y, Chen Z, et al. Sequencing and de novo assembly of a near complete indica rice genome. Nature Communications. 2017;8:15324.
7. Jennings DL. Raspberries and blackberries: their breeding, diseases and growth. Academic press; 1988.
8. Dossett M, Bassil NV, Lewers KS and Finn CE. Genetic diversity in wild and cultivated black raspberry (Rubus occidentalis L.) evaluated by simple sequence repeat markers. Genetic resources and crop evolution. 2012;59 8:1849-65.
9. Dossett M, Lee J and Finn CE. Inheritance of phenological, vegetative, and fruit chemistry traits in black raspberry. Journal of the American Society for Horticultural Science. 2008;133 3:408-17.
10. VanBuren R, Bryant D, Bushakra JM, Vining KJ, Edger PP, Rowley ER, et al. The genome of black raspberry (Rubus occidentalis). The Plant Journal. 2016;87 6:535-47.
11. Jibran R, Dzierzon H, Bassil N, Bushakra JM, Edger PP, Sullivan S, et al. Chromosome-scale scaffolding of the black raspberry (Rubus occidentalis L.) genome based on chromatin interaction data. Horticulture research. 2018;5 1:8.
12. Koren S, Walenz BP, Berlin K, Miller JR, Bergman NH and Phillippy AM. Canu: scalable and accurate long-read assembly via adaptive k-mer weighting and repeat separation. bioRxiv. 2017:071282.
13. Walker BJ, Abeel T, Shea T, Priest M, Abouelliel A, Sakthikumar S, et al. Pilon: an integrated tool for comprehensive microbial variant detection and genome assembly improvement. PloS one. 2014;9 11:e112963.
14. Finn CE, Lee J, VanBuren R, Bassil NV, Bryant DW, Gilmore BS, et al. A genetic linkage map of black raspberry (Rubus occidentalis) and the mapping of Ag (4) conferring resistance to the aphid Amphorophora agathonica. 2015.
15. English AC, Richards S, Han Y, Wang M, Vee V, Qu J, et al. Mind the gap: upgrading genomes with Pacific Biosciences RS long-read sequencing technology. PloS one. 2012;7 11:e47768.
16. Campbell MS, Law M, Holt C, Stein JC, Moghe GD, Hufnagel DE, et al. MAKER-P: a tool kit for the rapid creation, management, and quality control of plant genome annotations. Plant physiology. 2014;164 2:513-24.
17. Pertea M, Pertea GM, Antonescu CM, Chang T-C, Mendell JT and Salzberg SL. StringTie enables improved reconstruction of a transcriptome from RNA-seq reads. Nature biotechnology. 2015;33 3:290-5.

18. Lamesch P, Berardini TZ, Li D, Swarbreck D, Wilks C, Sasidharan R, et al. The Arabidopsis Information Resource (TAIR): improved gene annotation and new tools. Nucleic acids research. 2011;40 D1:D1202-D10.
19. Simão FA, Waterhouse RM, Ioannidis P, Kriventseva EV and Zdobnov EM. BUSCO: assessing genome assembly and annotation completeness with single-copy orthologs. Bioinformatics. 2015;31 19:3210-2.
20. VanBuren R, Bryant D, Edger PP, Tang H, Burgess D, Challabathula D, et al. Single-molecule sequencing of the desiccation-tolerant grass Oropetium thomaeum. Nature. 2015.
21. Jarvis DE, Ho YS, Lightfoot DJ, Schmöckel SM, Li B, Borm TJ, et al. The genome of Chenopodium quinoa. Nature. 2017;542 7641:307-12.
22. Xiang Y, Huang C-H, Hu Y, Wen J, Li S, Yi T, et al. Evolution of Rosaceae fruit types based on nuclear phylogeny in the context of geological times and genome duplication. Molecular biology and evolution. 2016;34 2:262-81.
23. Paterson AH, Bowers JE, Bruggmann R, Dubchak I, Grimwood J, Gundlach H, et al. The Sorghum bicolor genome and the diversification of grasses. Nature. 2009;457 7229:551.
24. Ohno S. Other Mechanisms for Achieving Gene Duplication. Evolution by Gene Duplication. Springer; 1970. p. 107-10.
25. Zhang HB, Zhao X, Ding X, Paterson AH and Wing RA. Preparation of megabase-size DNA from plant nuclei. The Plant Journal. 1995;7 1:175-84.
26. Langmead B and Salzberg SL. Fast gapped-read alignment with Bowtie 2. Nature methods. 2012;9 4:357-9.
27. McKenna A, Hanna M, Banks E, Sivachenko A, Cibulskis K, Kernytsky A, et al. The Genome Analysis Toolkit: a MapReduce framework for analyzing next-generation DNA sequencing data. Genome research. 2010;20 9:1297-303.
28. Li H and Durbin R. Fast and accurate short read alignment with Burrows–Wheeler transform. Bioinformatics. 2009;25 14:1754-60.
29. Bickhart DM, Rosen BD, Koren S, Sayre BL, Hastie AR, Chan S, et al. Single-molecule sequencing and chromatin conformation capture enable de novo reference assembly of the domestic goat genome. Nature Genetics. 2017;49 4:643-50.
30. Ou S and Jiang N. LTR_retriever: a highly accurate and sensitive program for identification of long terminal-repeat retrotransposons. Plant Physiology. 2017:pp. 01310.2017.
31. Bolger AM, Lohse M and Usadel B. Trimmomatic: a flexible trimmer for Illumina sequence data. Bioinformatics. 2014:btu170.
32. Dobin A, Davis CA, Schlesinger F, Drenkow J, Zaleski C, Jha S, et al. STAR: ultrafast universal RNA-seq aligner. Bioinformatics. 2013;29 1:15-21.
33. Love MI, Huber W and Anders S. Moderated estimation of fold change and dispersion for RNA-seq data with DESeq2. Genome biology. 2014;15 12:550.
34. Kolde R. Pheatmap: pretty heatmaps. R package version. 2012;61.
35. Tang H, Wang X, Bowers JE, Ming R, Alam M and Paterson AH. Unraveling ancient hexaploidy through multiply-aligned angiosperm gene maps. Genome research. 2008;18 12:1944-54.
36. VanBuren R, Wai CM, Colle M, Wang J, Sullivan S, Bushakra JM et al. Supporting data for "A near complete, chromosome-scale assembly of the black raspberry (Rubus occidentalis) genome" GigaScience Database.2018. http://dx.doi.org/10.5524/100465.

Figures:

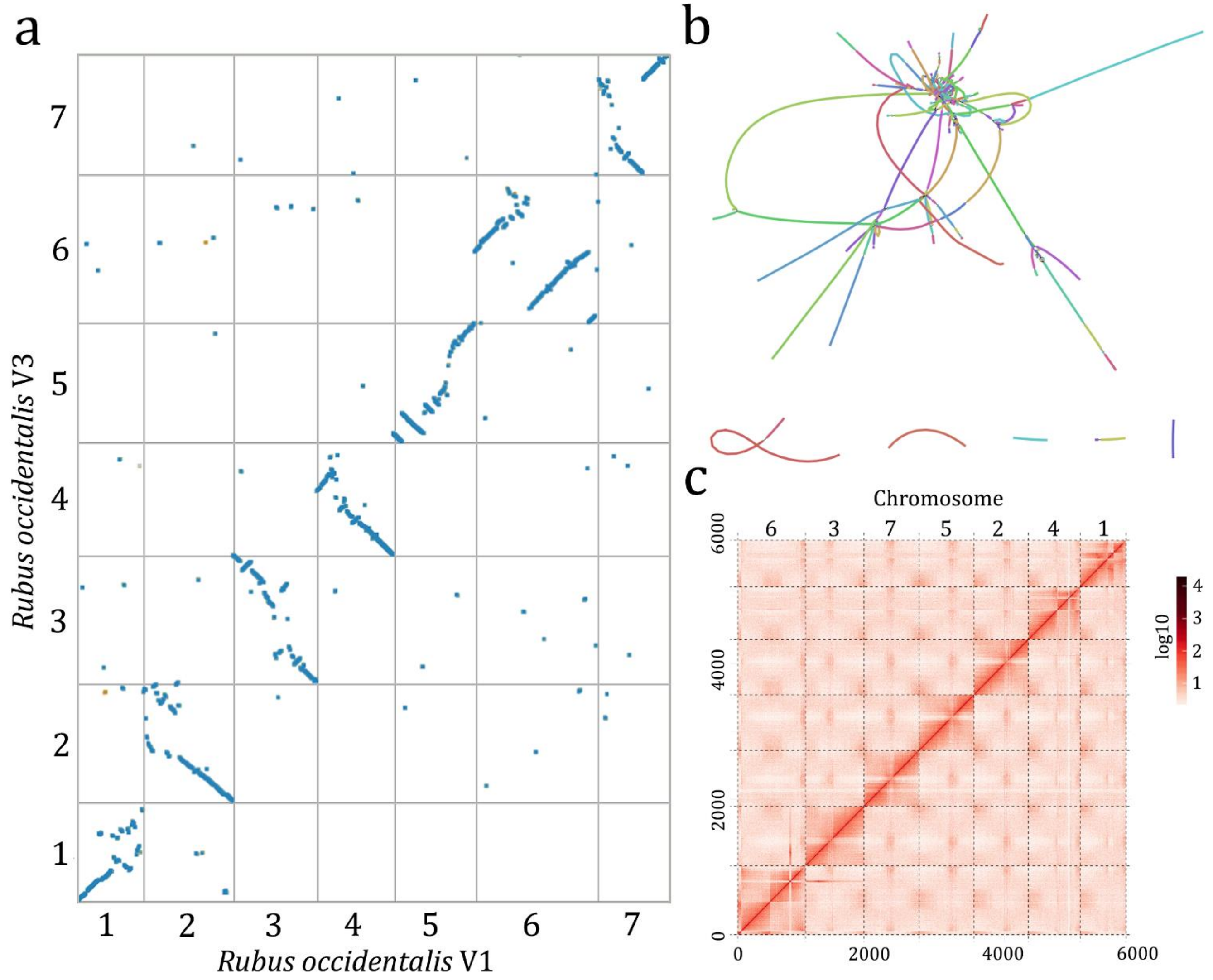

Figure 1. Updated chromosome scale assembly of black raspberry. (a) Syntenic dotplot of the black raspberry V1 and V3 assemblies. Each blue point denotes a collinear genomic region. (b) Assembly graph of the V3 reference. Each line (node) represents a contig in the Canu assembly and connections (edges) between contigs represent ambiguities in the graph structure. The color of contigs is randomly assigned. (c) Post-clustering heat map showing density of Hi-C interactions between contigs from the Proximity Guided Assembly.

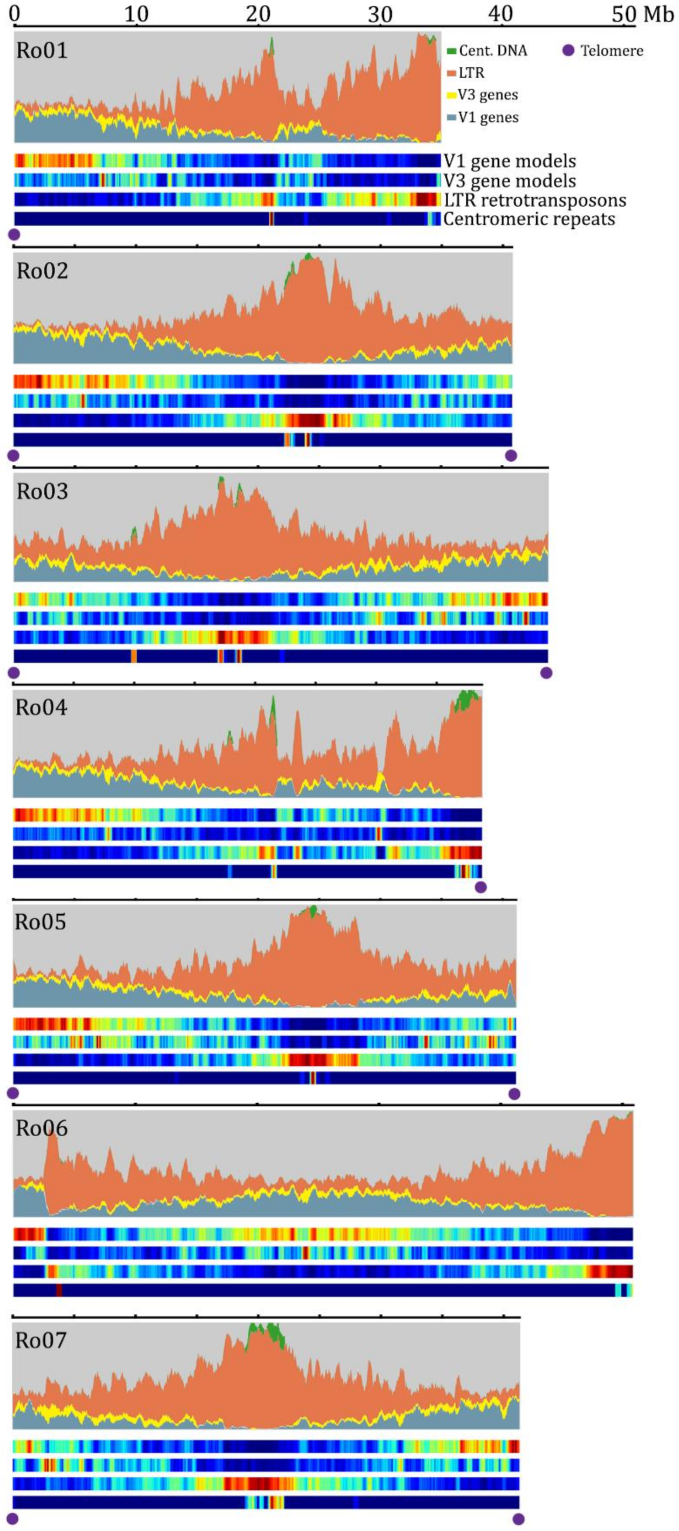

Figure 2: Genome landscape of the black raspberry V3 genome. The composition of long terminal repeat retrotransposons (LTRs), centromeric repeat arrays (Cent. DNA), gene models carried over from the V1 assembly, and new gene models in V3 are plotted in 50 kb bins with a 25kb sliding window. Terminal telomeric repeats are denoted by purple dots.

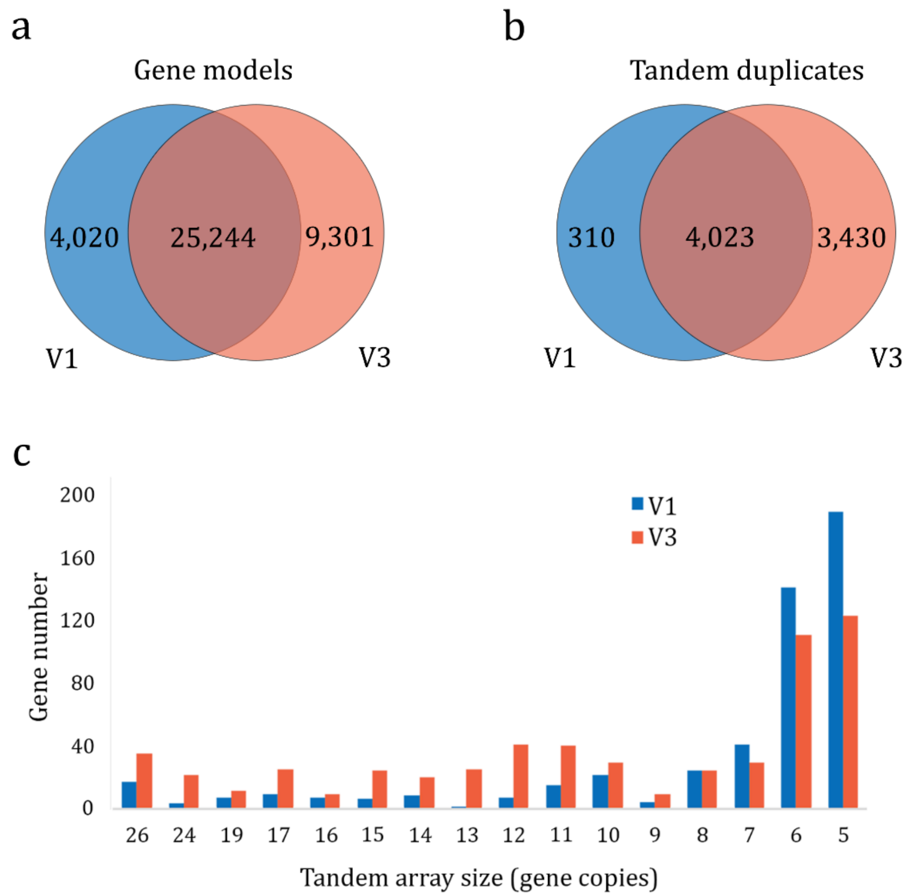

Figure 3. Comparison of tandem gene array sizes in the V1 and V3 black raspberry assemblies. (a) Venn diagram of gene models specific to V1 (blue), specific to V3 (orange) and shared. (b) Comparison of total tandem gene duplicates in V1 and V3. (c) The number of genes found in both the V1 and V3 assemblies (Blue) or only V3 (orange) is plotted for tandem arrays ranging in size from 5-26 copies. Array size is based on the V3 annotation.

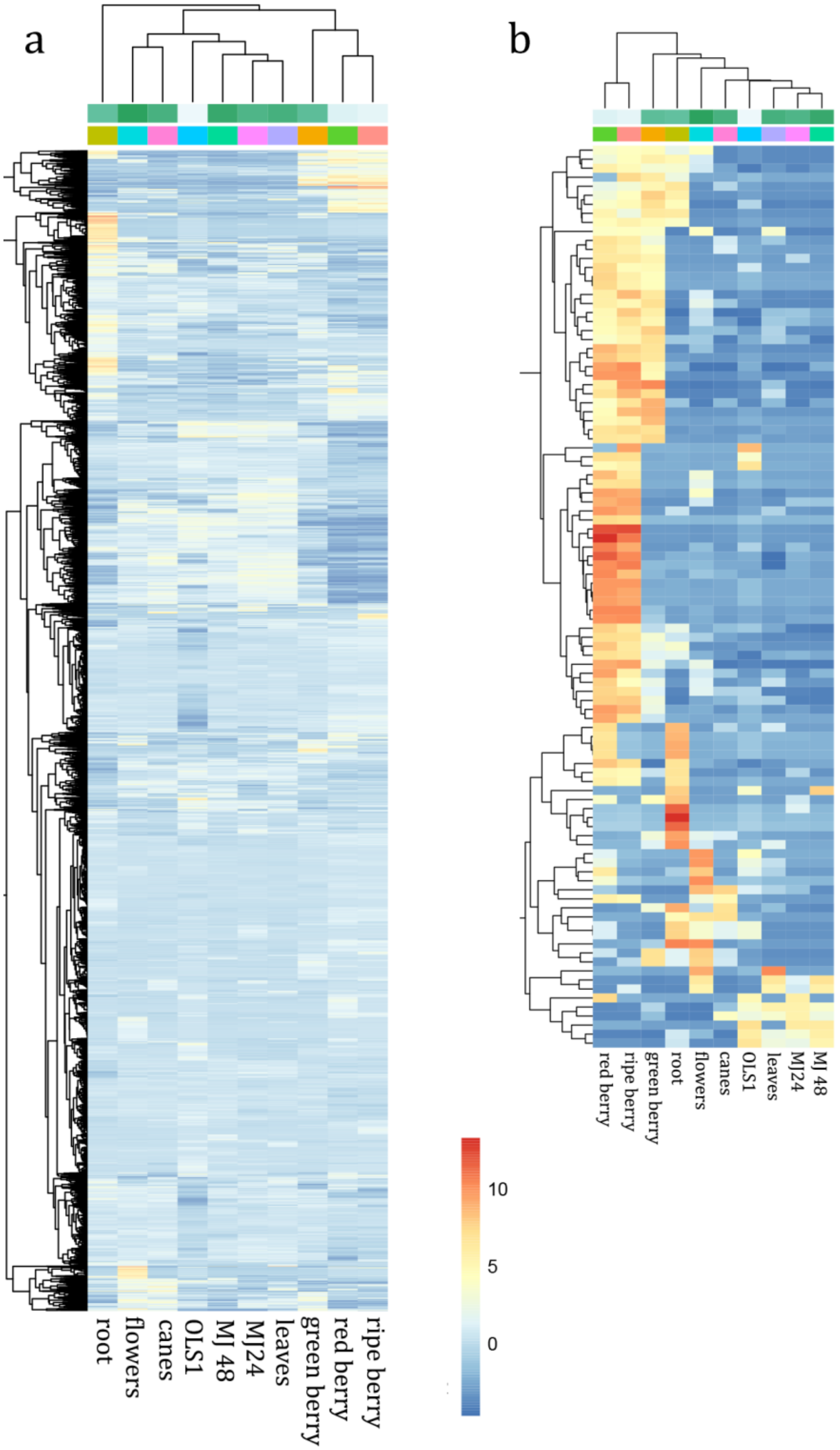

Figure 4. Expression patterns of new genes in the V3 black raspberry assembly. (a) Heatmap of expression patterns of all 6,070 new genes with detectable expression. (b) Expression patterns of the top 100 genes with highest expression. Blue indicates low expression and red indicates high expression. Expression values are plotted as log2 transformed FPKM.

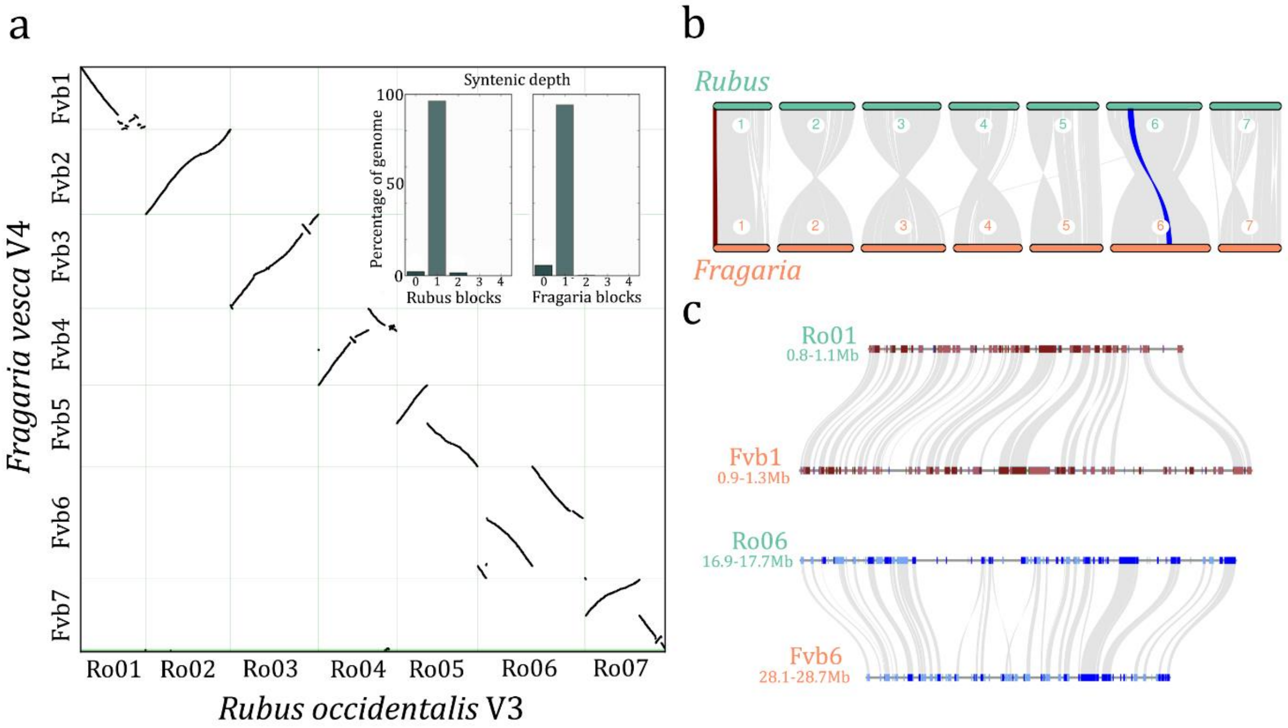

Figure 5. Comparative genomics of the black raspberry V3 and woodland strawberry (*Fragaria vesca*) V4 genomes. (a) Macrosyntenic dot plot between the black raspberry and *F. vesca* genomes. Each black dot represents a syntenic region between the two genomes. The inlaid bar graph shows syntenic depth of each red raspberry and *F. vesca* syntenic block. (b) Chromosome scale collinearity between black raspberry and *F. vesca*. The red collinear regions between Ro01 and Fvb1 blue regions between are Ro06 and Fvb6 shown in more detail in c. (c) Microsynteny of two regions showing lineage specific expansion in Fvb1 (top comparison) and Ro06 (bottom). Genes are shown in red or blue (top and bottom respectively) with colors indicating gene orientation (light are forward, dark are reverse). Syntenic gene pairs are connected by gray lines.

Table 1. Comparison of the black raspberry V1 and V3 assemblies

	V1	V3
**Total assembly size**	243 Mb	290 Mb
**Number of contigs**	11,936	235
**Number of scaffolds**	2,226	7
**Contig N50**	33.1 kb	5,100 kb
**Scaffold N50**	0.35 Mb	41.1 Mb
**LTR composition (%)**	16.20%	32.60%
**Number of genes**	28,005	34,545

Table 2. Summary of chromosome anchoring using the HiC genome map.

Chromosome	Anchored contigs	Total size (bp)
Ro01	19	34,302,027
Ro02	19	40,757,823
Ro03	30	43,767,452
Ro04	30	38,746,748
Ro05	25	41,095,993
Ro06	37	50,854,034
Ro07	75	41,277,220
**Total**	**235**	**290,801,297**

Click here to access/download
Supplementary Material
Supplement_3-22-18.docx
