# Supplementary material for: A near complete, chromosome-scale assembly of the black raspberry (Rubus occidentalis) genome
Source: Gigascience. 2018 Aug 9;7(8):giy094. doi: 10.1093/gigascience/giy094 (PMC6131213; doi:10.1093/gigascience/giy094)
Supplement: Reviewer_2_Original_Submission_(attachment).pdf [file giy094_reviewer_2_original_submission_(attachment).pdf]

This new black raspberry assembly is a nice improvement upon an existing resource. Chromosome level assemblies are particularly useful in breeding efforts and comparative genomics and are just easier to work with overall. The manuscript also provides some examples of genomic features that tend to be better assembled in PacBio assemblies versus Illumina. The manuscript was well written, clear, and concise. I have a few comments:

1) For Pilon correction, what was the rationale for 2 rounds of correction? It may be be useful to run more rounds until it reaches a plateau. Also, it wasn't clear if these were PE or SE Illumina reads.
2) I was curious if other parameters for Canu or other assemblers, such as Falcon, were also attempted in efforts to assemble the PacBio data. It can be good to run several assemblies with the corrected reads to find the optimal one. If you have this data it would be nice to include it.
3) What was the average size and range of sizes of gaps in the assembly? A supplemental graph could show this.
4) It is stated that there were several misassemblies in the V1 genome compared to V3. How do you know that none of these are errors in V3? Is it possible to use the existing PacBio, Illumina reads, or genetic maps to try to confirm this?
5) What is meant by "high confidence" gene models? Did you use AED scores or something else to establish this?
6) I think supplemental figure 2 could be included in the manuscript. If you do this, you should have a scale to explain how the colors correspond to values and label the gene names in panel b. I would also turn off clustering and keep the samples in the same order. Also, what are you plotting here, FPKM, log CPM?
7) Please provide your Canu configuration file as a supplement and also the results of the Augustus training (sensitivity/specificity). Did you provide your training set to Mario Stanke?
8) I would like to see more comparison to the V1 annotation. How many genes overlap, how many are new, how many were not found (you could make a Venn Diagram here). Also, are the gene lengths comparable between the two annotations, or do you see longer gene lengths in your annotation due to the better assembly? Or are they shorter meaning some residual indels may be breaking models?
9) Figure 2 would be clearer if the data was plotted on separate tracks. This could look nice as a Circos figure.
10) For table one, please keep the same unit across the row for contig N50. Also, what is the total assembly length and total repeat%?
