# Supplementary material for: A near complete, chromosome-scale assembly of the black raspberry (Rubus occidentalis) genome
Source: Gigascience. 2018 Aug 9;7(8):giy094. doi: 10.1093/gigascience/giy094 (PMC6131213; doi:10.1093/gigascience/giy094)
Supplement: Supplemental Tables and Figures [file giy094_supplemental_tables_and_figures.docx]

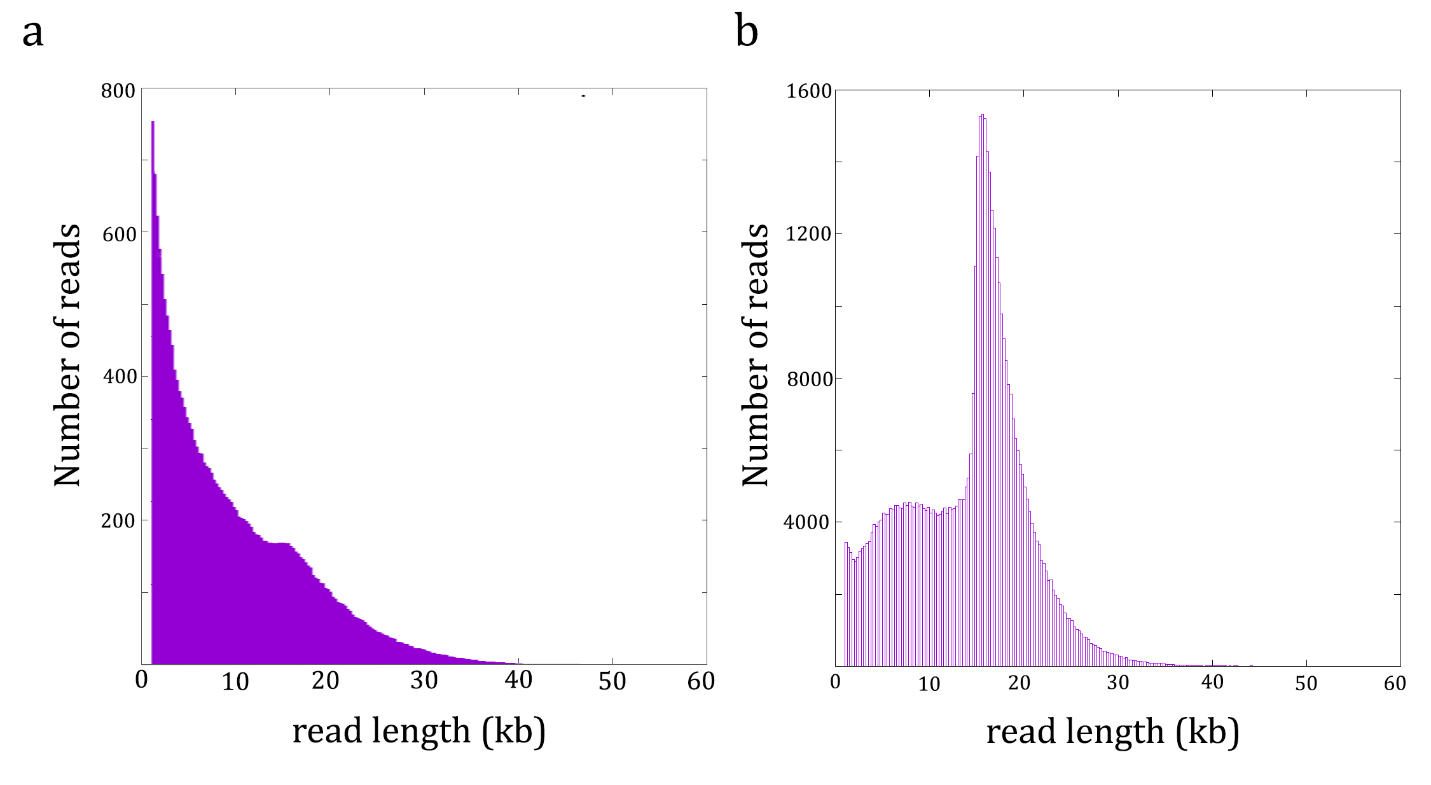


**Supplemental Figure 1**. Read length distribution of raw and error corrected PacBio reads. (a) Histogram of raw reads plotted in 100 bp window. (b) Histogram of error corrected reads in 250 bp windows.


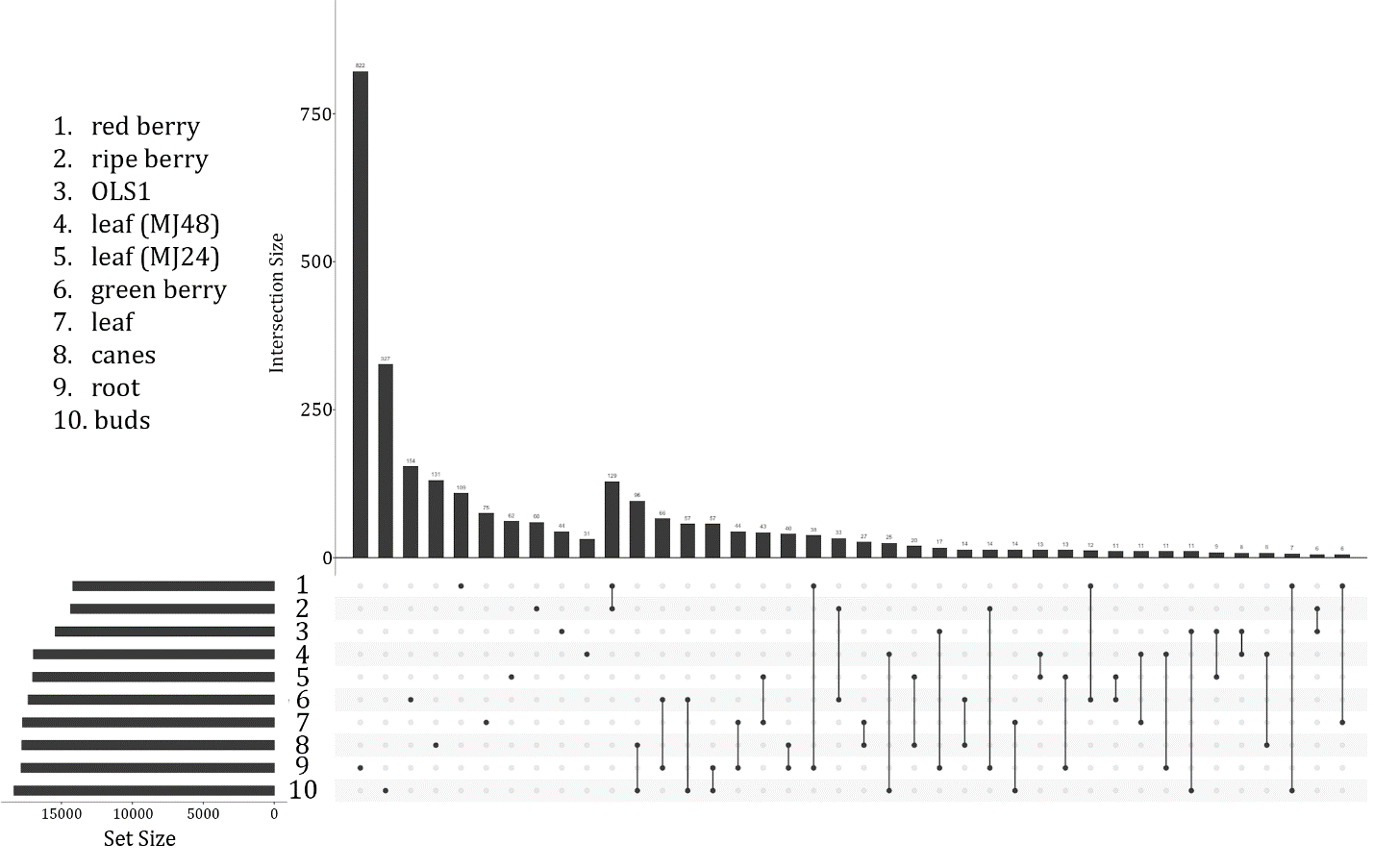


Supplemental Figure 2. Intersection analysis of the black raspberry gene expression analysis.

**Supplemental Table 1.** Summary of centromeric repeat arrays

| **Chromosome** | **Number of repeat monomers**  **(317 bp)** | **Start of centromeric array (bp)** | **End of centromeric repeat array (bp)** | **Putative centromere size (bp)** |
| --- | --- | --- | --- | --- |
| Ro01 | 110 | 20,621,883 | 20,795,294 | 173,411 |
| Ro02 | 200 | 22,294,763 | 25,177,654 | 2,882,891 |
| Ro03 | 160 | 16,788,196 | 21,996,483 | 5,208,287 |
| Ro04 | 1,204 | 17,818,823 | 21,684,906 | 3,866,083 |
| Ro05 | 234 | 23,616,090 | 25,706,595 | 2,090,505 |
| Ro07 | 1,146 | 19,079,686 | 22,000,322 | 2,920,636 |
| **Total** | **3,054** |  |  | **17,141,813** |

**Supplemental Table 2.** Expanded tandem gene arrays in the black raspberry genome compared to *F. vesca*.

| **Representative gene model in array** | **Tandem array size (Rubus)** | **Tandem array size (Fragaria)** | **Annotation** |
| --- | --- | --- | --- |
| Ro02_G21494 | 26 | 1 | S-acyltransferase |
| Ro02_G24232 | 26 | 1 | NBS-LRR |
| Ro01_G26393 | 19 | 1 | Hypothetical protein |
| Ro03_G16148 | 17 | 1 | Protein tyrosine kinase |
| Ro07_G23161 | 16 | 1 | amidohydrolase YtcJ isoform X2 |
| Ro04_G14171 | 15 | 1 | hypothetical protein RF2 |
| Ro04_G37141 | 15 | 1 | ATPase family associated |
| Ro06_G28872 | 13 | 1 | F-box protein |
| Ro03_G05878 | 12 | 1 | ABC transporter |
| Ro04_G36397 | 12 | 1 | Unknown protein |
| Ro05_G31112 | 12 | 1 | Membrane transport protein |
| Ro05_G14507 | 11 | 1 | Protein kinase |
| Ro05_G22172 | 11 | 1 | tRNA synthetase |
| Ro05_G31656 | 11 | 1 | pathogenesis-related protein |
| Ro01_G01090 | 10 | 1 | NB-ARC domain |
| Ro02_G21464 | 10 | 1 | O-methyltransferase |
