# Supplementary material for: A near complete, chromosome-scale assembly of the black raspberry (Rubus occidentalis) genome
Source: Gigascience. 2018 Aug 9;7(8):giy094. doi: 10.1093/gigascience/giy094 (PMC6131213; doi:10.1093/gigascience/giy094)
Supplement: Response_to_Reviewer_Comments_Original_Submission.pdf [file giy094_response_to_reviewer_comments_original_submission.pdf]

Author's Response To Reviewer Comments

Close

Reviewer reports:
Reviewer #1: This manuscript presents a significantly improved genome assembly (and annotation) of the black raspberry (Rubus occidentalis). I am in line with the authors, that the new assembly will improve trait mapping for breeding, and other functional genomics applications, especially in regions of high complexity. However, I have several concerns that the authors should address prior to publication of the manuscript:

1) I consider the golden standard approach to polish any long read assembly to be a combination of long read polishing via Quiver or Arrow and short read polishing via Pilon. Authors only applied the latter, likely removing the majority of shorter mis-assemblies. However larger errors likely remained. The authors should add that their assembly might still contain larger erroneous regions which are not corrected by Pilon's short read approach.

R: In our personal experience, we have run into problems with misassemblies around tandem repeats and LTRs using Arrow, so we typically won't use the PacBio data for error correction. HiC scaffolding failed to identify any misassembled contigs, supporting there are no large-scale errors still in the assembly. We agree with the reviewer that this could be a problem in our final assembly, and we have included discussion of possible misassemblies to the methods.

2) A detailed description of the methods used to define centromere sizes is missing. The authors should clearly lay out how the three lines of evidence, presence of centromeric arrays, repetitive element density, and Hi-C-based intra-chromosomal interactions were used to estimate the size of the centromere on each chromosome. Further they should investigate how much centromeric arrays / elements are in the unassembled reads that Canu usually returns, and speculate if their distribution mimics their assembled observation or could explain the missing centromeric repeats on Ro06.

R: It's difficult to accurately define the borders of centromeres without cytological evidence, so the numbers reported here are rough estimates. Centromeres were identified using three lines of evidence: 1) reduced intrachromosomal interactions in the Hi-C heat map, 2) increased density of LTR retrotransposons, 3) presence of centromere specific tandem repeat arrays (317 bp). First, the intrachromosomal interactions were used to locate the putative centromere locations. Centromere locations were validated by overlap with centromere specific tandem repeat arrays. The estimated borders of centromeres were identified by the presence of centromere specific tandem repeats and LTR retrotransposon density > 85%. We have added this to the methods section.
The unassembled reads file from Canu contains 65,252 reads collectively spanning 569 Mb. Within the unassembled read file, only 9 reads (spanning 75kb) contain at least two copies of the centromeric repeat array. This suggests the centromeres are well assembled in black raspberry. These reads may correspond to the centromere in Ro06 or this centromere may contain no centromere specific repeat arrays.

3) BUSCO should not be used to assess whole "transcriptome" annotation quality per se but only to estimate completeness of a genome assembly. The authors should instead use http://hibberdlab.com/transrate to benchmark their annotation by re-mapping their RNA-seq

reads to the old and the new reference, respectively the predicted transcripts.

R: We tried to run transrate (v1.0.3) but ran into several issues with the pipeline that are still unresolved based on discussion on GitHub. The transrate pipeline simply remaps RNAseq reads to the transcripts and references and outputs quality based metrics. This is a somewhat circular approach as the mapped RNAseq reads were used as transcript evidence for MAKER, so this approach could incorrectly inflate confidence in the annotation/assembly accuracy.
Roughly 96% of RNAseq reads map to the new assembly and the vast majority of these reads lie in predicted transcripts. Calculating the number of read mapping to transcripts is problematic as lncRNAs and TE are also expressed but were filtered from the annotation.

4) A definition of what the authors mean with tissue-specific expression pattern is missing. Both methods and results section should contain such a definition (including a definition of what is expressed and what is not). Results should be visualized using an intersection analysis, summarizing shared and tissue-specific expression patterns e.g., with a simple UpSetR plot.

R: Tissue specific expression is defined as having FPKM >1 in one tissue and FPKM < 1 for all other tissues. We have included this definition in the methods section. We have included an intersection analysis to summarize tissue specific and shared expression (see supplemental Figure 2).

5) The authors should add details about what the lineage specific expansion in Fvb1 (top comparison) and Ro06 (bottom) "contains". Is it linked 6) ?

R: These two panels represent two unrelated examples of regions with divergent gene composition between Rubus and Fragaria and are likely caused by a combination of lineage specific expansions/deletions in both genes and repetitive elements. We updated the text to discuss what these lineage specific regions likely contain (see lines 174-178):
"The black raspberry and F. vesca genomes are similar in size (290 vs. 240 Mb, respectively) and each genome has unique patterns of expansion/deletion based on gene-level microsynteny (Figure 4c). Differences in gene composition between species are likely due to a combination tandem gene duplications, retrotransposon mediated duplication/movement, fractionation/deletion, and mis-annotation. Expansion/deletion outside of genic regions is likely related to differences in repetitive element composition."

6) The expanded NLR cluster is quite an interesting finding. Authors should add some more details about the "locus". Please classify the 26 genes according into TNL (TIR domain present), CNL (coiled-coiled domain present) or RNL (RPW8 domain present). Shortly elaborate on the differences between the 26 transcripts (if there are any) as well as on the expression of the transcripts. What is the tandem array status in the other Rosaceae? It would be further interesting to carry out a codeml analysis to test for lineage- and site-specific adaptive changes considering the F. vesca copy as outgroup (in case the single copy status is common in the Rosaceae).

R: We agree with the reviewer that the large array of NBS-LRR proteins is interesting, and we speculate that similarly large tandem arrays are misassembled in other genomes. These analyses are beyond the scope of this manuscript and we have removed this sentence for incorporation into future work.

8) The last paragraph, I consider it sort of a mix between discussion and summary, should be tailored towards future comparative analysis within the Rosaceae, how the new black raspberry genome can contribute or how it will help to "expedite the development of improved black and red raspberry, blackberry and other Rubus cultivars" (taken from the first genome paper) in detail. Please remove the "finishing draft assemblies is important pitch" (everyone knew that when the first puzzly Illumina assemblies were released) and rewrite the whole paragraph.

R: We agree with the reviewer and have restructured the summary paragraph to better showcase the utility of the V3 black raspberry assembly.

Reviewer #2:

This new black raspberry assembly is a nice improvement upon an existing resource. Chromosome level assemblies are particularly useful in breeding efforts and comparative genomics and are just easier to work with overall. The manuscript also provides some examples of genomic features that tend to be better assembled in PacBio assemblies versus Illumina. The manuscript was well written, clear, and concise. I have a few comments:

1) For Pilon correction, what was the rationale for 2 rounds of correction? It may be be useful
to run more rounds until it reaches a plateau. Also, it wasn’t clear if these were PE or SE Illumina reads.

R: For most PacBio based genome projects, we usually run Pilon for 4-5 rounds or until the polishing plateaus. In this case, the first round of Pilon polishing corrected 102,366 Indels and 2,900 SNPs and 4,563 Indels and 0 SNPs were corrected in the second round. We attribute the ease of polishing to the high coverage of PacBio data, high coverage of PE Illumina data (~80x), and relatively simple genome structure. The high quality annotation and similarity to the Illumina based genome support the genome was adequately polished. We used PE ~300bp insert Illumina data from the Illumina based genome project for Pilon based polishing.

2) I was curious if other parameters for Canu or other assemblers, such as Falcon, were also attempted in efforts to assemble the PacBio data. It can be good to run several assemblies with the corrected reads to find the optimal one. If you have this data it would be nice to include it.

R: Given the relative simplicity of this genome (low heterozygosity, small size, and no recent LTR bursts), assembly using default parameters in Canu yielded a high quality assembly. This is supported by the relatively simple graph structure (see figure 1) and congruency with the HiC based anchoring. We did modify the following parameters for Canu: minReadLength=2000, GenomeSize=290Mb, minOverlapLength=1000. In more complex genomes, we will change the corrected output coverage, error rate, and overlapping options to phase or collapse haplotypes/repetitive regions. In our experience, Falcon produces similar assembly metrics to Canu, but is more likely to collapse repetitive regions. Because of this, we typically will not run Falcon.

3) What was the average size and range of sizes of gaps in the assembly? A supplemental

graph could show this.

R: The assembly contains 222 gaps across seven chromosomes after PBJelly based gap filling. Given the nature of HiC data, it is difficult to accurately estimate the physical size of gaps between anchored contigs. Optical maps and mate pair based scaffolding can be used to estimate gap size, but differences in chromatin interaction rates across the genome make this challenging with HiC data. All of the gaps were arbitrarily set to 100bp in this assembly. PBJelly was used to fill five of the gaps, but the remaining gaps are still 100 bp (though this is likely an underestimation). We have included gap lengths in the methods section.

4) It is stated that there were several misassemblies in the V1 genome compared to V3. How
do you know that none of these are errors in V3? Is it possible to use the existing PacBio, Illumina reads, or genetic maps to try to confirm this?

R: We agree with the reviewer and this is a great point. Identifying small-scale misassemblies in the Illumina based assembly is challenging given the fragmented nature of V1. We assembled ~47 Mb of new sequences and annotated thousands of new genes in V3, so there are numerous differences between assemblies. Large-scale errors in the V1 assembly are easier to identify. Maker density in the V1 map was relatively low and there were likely fine resolution marker order issues in this map stemming from erroneous marker calls from the GBS data. Because of this, there were likely ordering/orientation issues in the V1 pseudomolecules. We are currently remapping and analyzing the GBS data using the V3 reference, but this is beyond the scope of this manuscript. The HiC data was able to anchor and orient all 235 contigs with high confidence and no obvious misassemblies were identified. It is certainly possible there are misassemblies in V3, but they will be difficult to identify without additional lines of evidence. Similar assembly issues in Illumina based genomes have been identified in strawberry, apple, maize, and others.

5) What is meant by “high confidence” gene models? Did you use AED scores or something else to establish this?

R: High confidence was simply referring to models with AED scores < 1 that passed the default filtering thresholds by MAKER. We have removed the phrase ‘high confidence’ as we did not do any post filtering.

6) I think supplemental figure 2 could be included in the manuscript. If you do this, you should
have a scale to explain how the colors correspond to values and label the gene names in panel b. I would also turn off clustering and keep the samples in the same order. Also, what are you plotting here, FPKM, log CPM?

R: We have moved supplemental figure 2 to the main text and included a scale (log2 FPKM). B contains too many genes to include individual names. We chose to leave the clustering on to showcase that subsets of genes have tissue specific expression, which might explain why they were missed in the first annotation. Since the initial annotation is based on RNAseq data from only three tissues, many tissue specific genes were probably missed from a lack of transcript based evidence.

7) Please provide your Canu configuration file as a supplement and also the results of the

Augustus training (sensitivity/specificity). Did you provide your training set to Mario Stanke?

R: We listed the Canu parameters under the genome assembly section of the methods. We did not provide the training set to Mario Stanke.

8) I would like to see more comparison to the V1 annotation. How many genes overlap, how
many are new, how many were not found (you could make a Venn Diagram here). Also, are the gene lengths comparable between the two annotations, or do you see longer gene lengths in your annotation due to the better assembly? Or are they shorter meaning some residual indels may be breaking models?

R: We have added a Venn diagram to Figure 3 to show the annotation differences in V1 vs V3. In total, 25,244 gene models are shared between V1 and V3, 9,301 are new or greatly improved in V3, and 4,020 gene models from V1 were removed in V3. This is likely an artifact of improved assembly of gene space and an improved annotation pipeline.
The average gene length is roughly the same between both versions with an average gene length of 3,165 bp in V1 and 3,220 in V3. This suggests residual errors are probably not an issue in our new assembly.

9) Figure 2 would be clearer if the data was plotted on separate tracks. This could look nice as
a Circos figure.

R: We wanted to include all of the tracks on the same plot to make comparisons easier. For instance, overlaying gene and LTR density helps to clearly show the location of centromeric and pericentromeric regions. Circos plots are a good suggestion, but personally we find linear layouts of each chromosome easier to interpret.

10) For table one, please keep the same unit across the row for contig N50. Also, what is the
total assembly length and total repeat%?

R: We have updated Table 1 to include the total assembly length and standardized units. We annotated LTR transposons but did not annotate other repetitive elements, so we only included LTRs in this table.

Close
