# Supplementary material for: A near complete, chromosome-scale assembly of the black raspberry (Rubus occidentalis) genome
Source: Gigascience. 2018 Aug 9;7(8):giy094. doi: 10.1093/gigascience/giy094 (PMC6131213; doi:10.1093/gigascience/giy094)
Supplement: Reviewer_1_Report_(Original_Submission) -- Felix Bemm — 2/26/2018 Reviewed [file giy094_reviewer_1_report_(original_submission).pdf]

Reviewer Report

Title: **A near complete, chromosome-scale assembly of the black raspberry (Rubus occidentalis) genome**

Version: **Original Submission** **Date:** 2/26/2018

Reviewer name: **Felix Bemm**

Reviewer Comments to Author:

This manuscript presents a significantly improved genome assembly (and annotation) of the black raspberry (Rubus occidentalis). I am in line with the authors, that the new assembly will improve trait mapping for breeding, and other functional genomics applications, especially in regions of high complexity. However, I have several concerns that the authors should address prior to publication of the manuscript:

1) I consider the golden standard approach to polish any long read assembly to be a combination of long read polishing via Quiver or Arrow and short read polishing via Pilon. Authors only applied the latter, likely removing the majority of shorter mis-assemblies. However larger errors likely remained. The authors should add that their assembly might still contain larger erroneous regions which are not corrected by Pilon's short read approach.

2) A detailed description of the methods used to define centromere sizes is missing. The authors should clearly lay out how the three lines of evidence, presence of centromeric arrays, repetitive element density, and Hi-C-based intra-chromosomal interactions were used to estimate the size of the centromere on each chromosome. Further they should investigate how much centromeric arrays / elements are in the unassembled reads that Canu usually returns, and speculate if their distribution mimics their assembled observation or could explain the missing centromeric repeats on Ro06.

3) BUSCO should not be used to assess whole "transcriptome" annotation quality per se but only to estimate completeness of a genome assembly. The authors should instead use http://hibberdlab.com/transrate to benchmark their annotation by re-mapping their RNA-seq reads to the old and the new reference, respectively the predicted transcripts.

4) A definition of what the authors mean with tissue-specific expression pattern is missing. Both methods and results section should contain such a definition (including a definition of what is expressed and what is not). Results should be visualized using an intersection analysis, summarizing shared and tissue-specific expression patterns e.g., with a simple UpSetR plot.

5) The authors should add details about what the lineage specific expansion in Fvb1 (top

comparison) and Ro06 (bottom) "contains". Is it linked 6) ?

6) The expanded NLR cluster is quite an interesting finding. Authors should add some more details about the "locus". Please classify the 26 genes according into TNL (TIR domain present), CNL (coiled-coiled domain present) or RNL (RPW8 domain present). Shortly elaborate on the differences between the 26 transcripts (if there are any) as well as on the expression of the transcripts. What is the tandem array status in the other Rosaceae? It would be further interesting to carry out a codeml analysis to test for lineage- and site-specific adaptive changes considering the F. vesca copy as outgroup (in case the single copy status is common in the Rosaceae).

8) The last paragraph, I consider it sort of a mix between discussion and summary, should be tailored towards future comparative analysis within the Rosaceae, how the new black raspberry genome can contribute or how it will help to "expedite the development of improved black and red raspberry, blackberry and other Rubus cultivars" (taken from the first genome paper) in detail. Please remove the "finishing draft assemblies is important pitch" (everyone knew that when the first puzzly Illumina assemblies were released) and rewrite the whole paragraph.

Methods

Are the methods appropriate to the aims of the study, are they well described, and are necessary controls included? Yes

Conclusions

Are the conclusions adequately supported by the data shown? Yes

Reporting Standards

Does the manuscript adhere to the journal's guidelines on minimum standards of reporting? Yes

Choose an item.

Statistics

Are you able to assess all statistics in the manuscript, including the appropriateness of statistical tests used? Yes, and I have assessed the statistics in my report.

Quality of Written English

Please indicate the quality of language in the manuscript: Needs some language corrections before being published

Declaration of Competing Interests

Please complete a declaration of competing interests, considering the following questions:

- Have you in the past five years received reimbursements, fees, funding, or salary from an organisation that may in any way gain or lose financially from the publication of this manuscript, either now or in the future?
- Do you hold any stocks or shares in an organisation that may in any way gain or lose financially from the publication of this manuscript, either now or in the future?
- Do you hold or are you currently applying for any patents relating to the content of the manuscript?
- Have you received reimbursements, fees, funding, or salary from an organization that holds or has applied for patents relating to the content of the manuscript?
- Do you have any other financial competing interests?
- Do you have any non-financial competing interests in relation to this paper?

If you can answer no to all of the above, write 'I declare that I have no competing interests' below. If your reply is yes to any, please give details below.

I declare that I have no competing interests.

I agree to the open peer review policy of the journal. I understand that my name will be included on my report to the authors and, if the manuscript is accepted for publication, my named report including any attachments I upload will be posted on the website along with the authors' responses. I agree for my report to be made available under an Open Access Creative Commons CC-BY license (http://creativecommons.org/licenses/by/4.0/). I understand that any comments which I do not wish to be included in my named report can be included as confidential comments to the editors, which will not be published.

I agree to the open peer review policy of the journal

To further support our reviewers, we have joined with Publons, where you can gain additional credit to further highlight your hard work (see: https://publons.com/journal/530/gigascience). On publication of this paper, your review will be automatically added to Publons, you can then choose whether or not to claim your Publons credit. I understand this statement.

Yes Choose an item.
