# Supplementary material for: A near complete, chromosome-scale assembly of the black raspberry (Rubus occidentalis) genome
Source: Gigascience. 2018 Aug 9;7(8):giy094. doi: 10.1093/gigascience/giy094 (PMC6131213; doi:10.1093/gigascience/giy094)
Supplement: Reviewer_1_Report_(Revision_1) -- Felix Bemm — 5/15/2018 Reviewed [file giy094_reviewer_1_report_(revision_1).pdf]

Reviewer Report

Title: **A near complete, chromosome-scale assembly of the black raspberry (Rubus occidentalis) genome**

Version: **Revision 1** **Date:** 5/15/2018

Reviewer name: **Felix Bemm**

Reviewer Comments to Author:

1) I consider the golden standard approach to polish any long read assembly to be a combination of long read polishing via Quiver or Arrow and short read polishing via Pilon. Authors only applied the latter, likely removing the majority of shorter mis-assemblies. However larger errors likely remained. The authors should add that their assembly might still contain larger erroneous regions which are not corrected by Pilon's short read approach.

R: In our personal experience, we have run into problems with misassemblies around tandem repeats and LTRs using Arrow, so we typically won't use the PacBio data for error correction. HiC scaffolding failed to identify any misassembled contigs, supporting there are no large-scale errors still in the assembly. We agree with the reviewer that this could be a problem in our final assembly, and we have included discussion of possible misassemblies to the methods.

RE: HiC scaffolding would not detect misassemblies on the lower kb ranges. It simply does not have enough resolution at a normal depth there. I am fine with the commment in the methods though.

3) BUSCO should not be used to assess whole "transcriptome" annotation quality per se but only to estimate completeness of a genome assembly. The authors should instead use http://hibberdlab.com/transrate to benchmark their annotation by re-mapping their RNA-seq reads to the old and the new reference, respectively the predicted transcripts.

R: We tried to run transrate (v1.0.3) but ran into several issues with the pipeline that are still unresolved based on discussion on GitHub. The transrate pipeline simply remaps RNAseq reads to the transcripts and references and outputs quality based metrics. This is a somewhat circular approach as the mapped RNAseq reads were used as transcript evidence for MAKER, so this approach could incorrectly inflate confidence in the annotation/assembly accuracy.
Roughly 96% of RNAseq reads map to the new assembly and the vast majority of these reads lie in predicted transcripts. Calculating the number of read mapping to transcripts is problematic as lncRNAs and TE are also expressed but were filtered from the annotation.

RE: Please change line 136 "We assessed annotation quality" to "We assessed annotation completeness"

6) The expanded NLR cluster is quite an interesting finding. Authors should add some more details about the "locus". Please classify the 26 genes according into TNL (TIR domain present), CNL (coiled-coiled domain present) or RNL (RPW8 domain present). Shortly elaborate on the differences between the 26 transcripts (if there are any) as well as on the expression of the transcripts. What is the tandem array status in the other Rosaceae? It would be further interesting to carry out a codeml analysis to test for lineage- and site-specific adaptive changes considering the F. vesca copy as outgroup (in case the single copy status is common in the Rosaceae).

R: We agree with the reviewer that the large array of NBS-LRR proteins is interesting, and we speculate that similarly large tandem arrays are misassembled in other genomes. These analyses are beyond the scope of this manuscript and we have removed this sentence for incorporation into future work.

RE: I am looking forward to that manuscript.

Methods

Are the methods appropriate to the aims of the study, are they well described, and are necessary controls included? Choose an item.

Conclusions

Are the conclusions adequately supported by the data shown? Choose an item.

Reporting Standards

Does the manuscript adhere to the journal's guidelines on minimum standards of reporting? Choose an item.

Choose an item.

Statistics

Are you able to assess all statistics in the manuscript, including the appropriateness of statistical tests used? Choose an item.

Quality of Written English

Please indicate the quality of language in the manuscript: Choose an item.

Declaration of Competing Interests

Please complete a declaration of competing interests, considering the following questions:

- Have you in the past five years received reimbursements, fees, funding, or salary from an organisation that may in any way gain or lose financially from the publication of this manuscript, either now or in the future?
- Do you hold any stocks or shares in an organisation that may in any way gain or lose financially from the publication of this manuscript, either now or in the future?
- Do you hold or are you currently applying for any patents relating to the content of the manuscript?
- Have you received reimbursements, fees, funding, or salary from an organization that holds or has applied for patents relating to the content of the manuscript?
- Do you have any other financial competing interests?
- Do you have any non-financial competing interests in relation to this paper?

If you can answer no to all of the above, write 'I declare that I have no competing interests' below. If your reply is yes to any, please give details below.

I declare that I have no competing interests

I agree to the open peer review policy of the journal. I understand that my name will be included on my report to the authors and, if the manuscript is accepted for publication, my named report including any attachments I upload will be posted on the website along with the authors' responses. I agree for my report to be made available under an Open Access Creative Commons CC-BY license (http://creativecommons.org/licenses/by/4.0/). I understand that any comments which I do not wish to be included in my named report can be included as confidential comments to the editors, which will not be published.

I agree to the open peer review policy of the journal

To further support our reviewers, we have joined with Publons, where you can gain additional credit to further highlight your hard work (see: https://publons.com/journal/530/gigascience). On publication of this paper, your review will be automatically added to Publons, you can then choose whether or not to claim your Publons credit. I understand this statement.

Yes Choose an item.
